# Cycloastragenol in inflammation-related diseases: mechanisms, pharmacokinetics, and translational prospects

**DOI:** 10.3389/fphar.2025.1732996

**Published:** 2026-01-12

**Authors:** Chun Zhao, Xiuhua Yang, Man Yao, Xiaoxuan Song, Jingtong Dai, Pilong He

**Affiliations:** 1 Sichuan Province Orthopedic Hospital, Chengdu, China; 2 Deyang Sixth People’s Hospital, Deyang, China; 3 Department of Ophthalmology, Chengdu Integrated TCM & Western Medicine Hospital Chengdu, Chengdu, China; 4 School of Pharmacy, Chengdu University of Traditional Chinese Medicine, Chengdu, China

**Keywords:** anti-inflammatory, cycloastragenol, disease intervention, inflammatory cytokines, natural products, NLRP3inflammasome

## Abstract

Chronic inflammation, driven by dysregulated immune responses and oxidative stress, underlies the pathogenesis of numerous diseases, from neurodegeneration to cancer. Cycloastragenol (CAG), a bioactive triterpenoid derived from Astragalus membranaceus, has emerged as a multifaceted therapeutic candidate due to its unique ability to simultaneously modulate inflammatory signaling networks, while exhibiting a favorable safety profile in preclinical models. This study aims to systematically evaluate the molecular mechanisms of CAG, including its coordinated anti-inflammatory, immune-regulatory, and tissue-protective effects. By integrating evidence from pharmacology, metabolomics, and clinical studies, our aim is to elucidate the therapeutic potential of CAG and identify strategies to overcome its pharmacokinetic limitations for clinical translation. A comprehensive literature review was conducted using databases such as PubMed, Web of Science, and Science Direct, employing target keywords related to cycloastragenol, inflammation, and disease treatment. Our analysis reveals that CAG exerts multidimensional and networked anti-inflammatory effects by synergistically regulating key inflammatory nodes such as NF-κB, Nrf2, and the NLRP3 inflammasome, as well as by alleviating oxidative stress. It has demonstrated therapeutic potential in diseases such as cancer, neurological disorders, asthma, and visceral fibrosis. CAG exerts significant anti-inflammatory effects by targeting the axis associated with inflammation, oxidative stress, and immune dysregulation. However, future efforts need to focus on improving its bioavailability and verifying its safety in human trials to develop a new generation of anti-inflammatory therapies.

## Graphical Abstract

**Figure:**
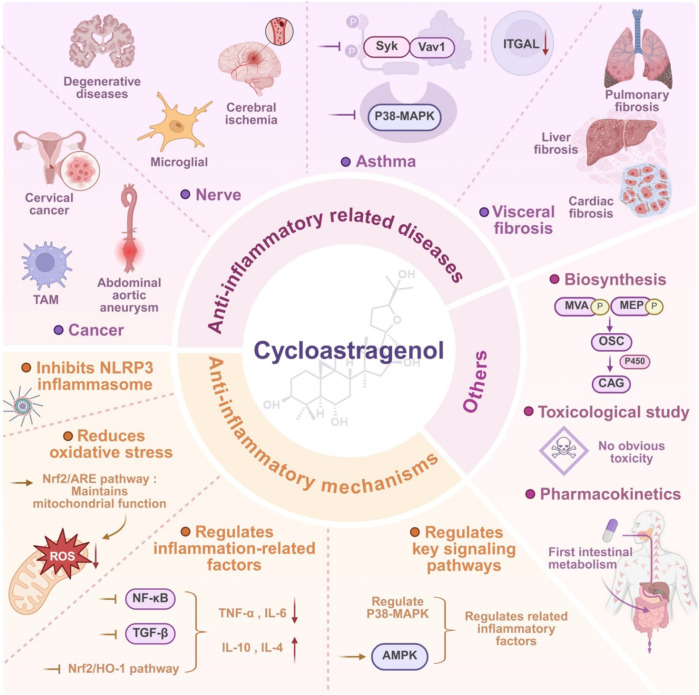
Graphical abstract: Cycloastragenol exerts multidimensional and networked anti-inflammatory effects by modulating multiple inflammatory nodes such as NF-κB, Nrf2, and the NLRP3 inflammasome, and by alleviating oxidative stress. It has shown therapeutic potential in diseases such as cancer, neurological disorders, asthma, and visceral fibrosis.

## 1 Introduction

The 21st century has witnessed an unprecedented recognition of inflammation as the common denominator underlying most chronic diseases that plague modern society. From cardiovascular disease and cancer to neurodegenerative disorders and metabolic syndrome, dysregulated inflammatory responses emerge as the central pathogenic mechanism (Alfaddagh et al., 2020; Amin et al., 2020; Chen et al., 2017). Despite this fundamental understanding, our therapeutic arsenal remains remarkably limited. In inflammatory bowel diseases, approximately 10%–30% of patients do not respond to initial treatment with TNFα antagonists, and 23%–46% of patients lose their response over time (Roda et al., 2016). This therapeutic gap represents one of the most pressing challenges in modern medicine, demanding innovative approaches that transcend the limitations of conventional single-target interventions (Zhu et al., 2025).

Current anti-inflammatory therapies face a fundamental paradox: while NSAIDs and glucocorticoids remain the cornerstone of treatment, their therapeutic efficacy is severely constrained by dose-limiting toxicities and mechanistic limitations (Oray et al., 2016; Zhang et al., 2006). NSAID, despite their widespread use, fails to address the upstream inflammatory cascades and carries significant cardiovascular and gastrointestinal risks (Bhala et al., 2013), with the probability of an increased risk of myocardial infarction associated with the use of an NSAID for one to 7 days being 92%–99% (Bally et al., 2017). GCs induce hyperglycemia and promote the development of type 2 diabetes by increasing hepatic gluconeogenesis and downregulating IRS-1 in fat and muscle, which causes insulin resistance (Reichardt et al., 2021).

This recognition has catalyzed a paradigm shift in anti-inflammatory drug discovery: from single-target blockade to network modulation. Systems biology analyzes reveal that sustainable inflammatory resolution requires simultaneous modulation of multiple key nodes within the inflammatory network (Chen et al., 2008). Natural products, evolved over millennia to interact with biological systems, inherently possess this multi-target capability (Zhao et al., 2025). Among these, triterpenoid saponins represent a particularly promising class, with their amphipathic structure enabling interaction with both membrane receptors and intracellular targets (Juang and Liang, 2020). In this context, Cycloastragenol (CAG) emerges as a molecular paradigm for next-generation anti-inflammatory therapies. Unlike conventional drugs constrained by their synthetic origins, CAG is a tetracyclic triterpenoid compound derived from *Astragalus membranaceus*, and it shows great potential to modulate multiple inflammatory nodes while maintaining cellular homeostasis (Yu et al., 2018). Recent evidence indicates CAG orchestrates a multi-pronged anti-inflammatory strategy: at the molecular level, by interfering with inflammasome assembly and cytokine release (Deng et al., 2019); at the signaling nexus, by fine-tuning critical pathways such as NF-κB and MAPK (Salminen et al., 2011), and at the cellular level, by influencing macrophage polarization and T-cell differentiation (Chen et al., 2022; Sun et al., 2017). This networked regulatory paradigm offers a distinct advantage in navigating the complexities of inflammatory diseases, potentially mitigating the activation of the compensatory pathway often seen with single-target agents (Fioranelli et al., 2021).

However, translating this therapeutic promise into clinical reality faces significant challenges that this review systematically addresses. Three critical knowledge gaps currently impede CAG’s clinical translation: initially, while CAG’s pleiotropic effects are well-documented, the hierarchical organization and temporal dynamics of its multitarget engagement remain poorly understood. Subsequently, the structure-activity relationships that govern its selective modulation of inflammatory pathways—critical for rational drug design—have not been systematically elucidated. Ultimately, the translational pathway from promising preclinical data to evidence-based clinical protocols remains undefined.

To date, reviews have extensively explored CAG’s mechanisms, progress, and applications of CAG in telomerase enhancement and macrophage polarization. However, research on the role of CAG’s in inflammation remains limited. Key gaps include its effects on inflammatory pathways, cytokine interactions, disease model interventions, and molecular mechanisms. In this context, this review addresses these gaps through a systematic analysis that moves from molecular mechanisms to clinical applications. We first establish CAG’s biosynthetic origins and metabolic fate, providing the foundation for understanding its bioavailability challenges. We then construct a comprehensive map of CAG’s anti-inflammatory network, integrating recent discoveries in inflammasome biology, metabolic-inflammatory crosstalk, and cellular senescence. Finally, we critically evaluate CAG’s therapeutic applications across multiple disease models, identifying both opportunities and obstacles for clinical translation. In an era where the limitations of reductionist drug discovery are increasingly apparent, CAG offers a compelling model for how evolutionary-optimized natural products can address the complexity of human inflammatory diseases.

## 2 Biosynthesis of cycloastragenol

CAG is a key active triterpenoid saponin in medicinal plants such as *A. membranaceus*, but its natural content is low, severely limiting large-scale production and clinical application. Therefore, elucidating and optimizing its biosynthetic pathway is of great importance for both basic research and drug development.

### 2.1 The cycloastragenol biosynthetic pathway: enzymatic cascade and metabolic bottlenecks

**FIGURE 1:**
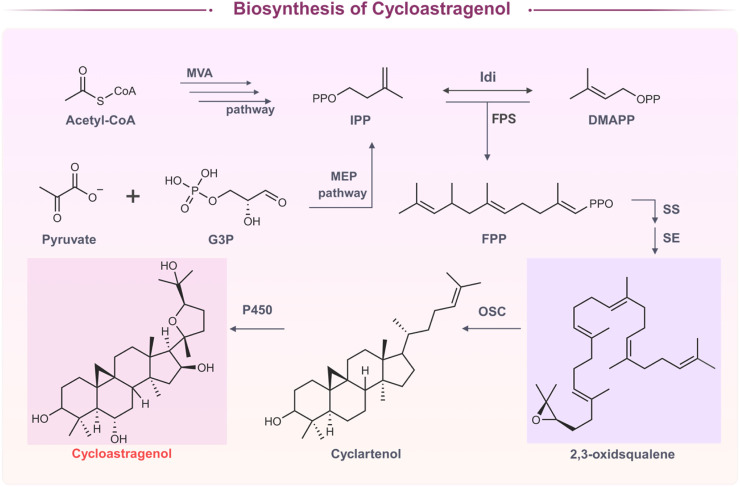
Biosynthesis of cycloastragenol.

The biosynthesis of CAG is a complex multi-step process involving enzymatic cascade reactions and a strict gene regulatory network. It begins with the mevalonate (MVA) and methylerythritol phosphate (MEP) pathways, which together generate isoprenoid diphosphate (IPP) and dimethylallyl diphosphate (DMAPP) (Pérez et al., 2022; Saito and Sawai, 2011). Under the catalysis of farnesyl diphosphate synthase (FPS), these two compounds condense to form farnesyl diphosphate (FPP), a core node metabolite in triterpenoid synthesis (Tian et al., 2020). It is worth noting that only a portion of FPP flows into the CAG pathway, with the remainder being consumed competitively in sterol synthesis (such as stigmasterol). This inefficient distribution of metabolic flux is one of the key reasons for the low natural yield (Valitova et al., 2024). FPP is then converted to 2,3-oxidosqualene by squalene synthase (SS) and squalene epoxidase (SE), and subsequently cyclized to intermediates such as α-amyrin and β-amyrin by oxidosqualene cyclase (OSC) (Chen et al., 2023; Tuan et al., 2015). The subsequent steps involve a series of oxidative modifications mediated by cytochrome P450 enzymes (CYP450), which introduce hydroxyl and epoxy groups into the cyclized intermediates to produce CAG (Figure 1) (Chen et al., 2023). In addition, Chen et al. found that OSC in the unique gene cluster III of *A. membranaceus* is involved in the diversification of triterpenoids (Chen et al., 2023). Although more than 80% of P450 genes are highly expressed in the roots of *A. membranaceus*, and 12 P450s are closely related to the biosynthesis of astragaloside IV (Wang et al., 2024b). However, the P450s involved in the biosynthesis of CAG, including phylogenetic analysis, expression profiles, and their relationship with CAG biosynthesis, have not been systematically studied yet, which is the focus and difficulty of current research. Advances in plant genomics and metabolic engineering have made it possible to identify and functionally characterize the key enzymes involved in CAG biosynthesis, such as cycloartenol synthase (CAS) and specific CYP450s (Tian et al., 2024). These findings provide a molecular basis for pathway reconstruction and yield improvement in heterologous systems. were found.

### 2.2 Strategies for cycloastragenol production: chemical synthesis and biocatalytic innovations

The *in vitro* synthesis of CAG is achieved mainly through two strategies: chemical synthesis or biocatalysis, aiming to overcome the problem of low natural extraction efficiency. Chemical synthesis typically starts with Astragaloside IV, involving multiple reaction steps to construct the unique cycloartane skeleton and the C-20,24-epoxy structure of CAG (Bedir et al., 2015). For example, cycloastragenol can be obtained from astragaloside IV through methods such as acid hydrolysis, Smith degradation, and enzymatic and microbial hydrolysis. However, this approach faces challenges such as overall low yields, environmental pollution, and the separation and purification of the product (Yuan et al., 2022). Feng et al. used Smith degradation to prepare cycloastragenol from astragaloside, and after optimizing the reagent dosage, the conversion yield of cycloastragenol could reach more than 80% (Feng et al., 2014).

Biocatalysis, on the other hand, utilizes microbial cell factories or enzyme cascade reactions *in vitro*, offering a greener and potentially more scalable alternative. For example, engineered yeast strains that express plant-derived CAS and CYP450 hydroxylases can convert squalene or cycloartenol into CAG. In 2022, Yuan et al. optimized CYP450 expression in yeast, increasing the production of CAG. The primary competitive pathway for CAS synthesis is the biosynthesis of lanosterol. Promoter engineering effectively regulates the expression of yeast genes, thereby inhibiting the lanosterol competitive pathway. However, the low specificity of structural modifications of key enzymes, such as CYP450s, in the downstream biosynthetic pathway of cycloastragenol limits its large-scale application (Yuan et al., 2022). The main bottlenecks in biotechnological production include the long transformation period and the high cost. These challenges are being addressed through the adoption of a rational gene extraction strategy to detect target enzymes, such as β-glucosidase (Cheng et al., 2020). Looking forward, the integration of synthetic biology and chemical semisynthesis is a promising trend. For example, microorganisms can be engineered to produce key intermediates (such as cycloartenol), which are then converted to the target product *via* short-step chemical modifications (Hage-Hülsmann et al., 2019). This hybrid approach aims to balance efficiency, scalability, and cost-effectiveness, paving the way for industrial-scale CAG production.

In brief, a comprehensive understanding of the biosynthetic pathway and advances in metabolic engineering and synthetic biology are essential to overcome current limitations in the supply of CAG. These efforts will not only facilitate their pharmaceutical development but also provide a model for the sustainable production of other valuable plant-derived natural products.

## 3 Pharmacokinetic profile and drug-drug interactions of cycloastragenol

Before applying it to preclinical studies, it is necessary to first understand its pharmacokinetic and metabolic characteristics to evaluate its therapeutic effects, safety, and clinical translation potential. This section systematically reviews the absorption, distribution, metabolism, excretion, and drug-drug interaction profiles of CAG, highlighting key challenges and future research directions.

### 3.1 Multi-dimensional pharmacokinetic characteristics of cycloastragenol

CAG is primarily absorbed in the intestine *via* passive diffusion across intestinal epithelial cells. Rat studies have shown that CAG is absorbed primarily in the small intestine, Caco-2 cell models confirming the presence of CAG metabolites on both the apical and basolateral sides, indicating first-pass intestinal metabolism during transepithelial transport (Zhu et al., 2010). The oral absorption of CAG is moderate, with a maximum plasma concentration (C_max_) typically observed approximately 2.06 h after administration and an elimination half-life (T_1/2_) of approximately 5.23 h in rats. Human studies suggest a longer elimination half-life, and both C_max_ and area under the curve (AUC) increase with dose, indicating dose-dependent pharmacokinetics. However, the oral bioavailability of CAG is relatively low. For example, rat studies have shown that the oral bioavailability of a dose of 10 mg/kg is only 25.70%, suggesting significant metabolic barriers and posing a challenge for clinical application (Ma et al., 2016). This low bioavailability is likely due to extensive first-pass metabolism and limited solubility, underscoring the need for innovative formulation strategies. The pharmacokinetic parameters of CAG in various models are summarized in Table 1.

**TABLE 1:** Pharmacokinetic information related to cycloastragenol.

Model	Dose	Administration route	C_max_	T_1/2_	T_max_	References
Male Sprague-Dawley rats	10, 20, and 40 mg/kg	In vivo	180.95 ± 48.01 ng/mL ∼ 566.67 ±71.66 ng/mL	5.23 ± 1.55 h ∼ 7.33 ± 3.03 h	1.48 ± 0.36 h∼2.35 ± 1.17 h	Ma et al. (2016)
​	10 mg/kg	In vivo	—	1.68 ± 0.34 h	—	​
Male Kunming mice	30 mg/kg	In vivo	474.9 ng/mL	0.32 h	8 h	He et al. (2018)
Human	35, 70, and 140 g/person	In vivo	2.22 ± 1.12 ng/mL ∼ 5.30 ± 3.61 ng/mL	20.71 ± 9.42 h ∼ 23.22 ± 10.71 h	21.00 ± 12.62 h ∼ 25.50 ± 9.72 h	Wang et al. (2021)
Male Sprague– Dawley rats	10 mg/kg 1.5 mg/kg	In vivo In vivo	9.67 ± 0.82 nM 8.33 ± 0.82 nM	— —	8.0 h 5.56 h	Jin et al. (2015)
Sprague-Dawley rats	18.84 g/kg Astragali Radix	In vivo	12.96 ± 7.28 ng/mL	11.17 ± 6.59 h	10.33 ± 1.51 h	Xiang et al. (2025)

After absorption, CAG is ubiquitously distributed in rats, with phase I metabolites 7, 6 and 1 identified in fecal, urinary, and biliary samples, respectively, but no phase II metabolites were detected (Ma et al., 2016). CAG undergoes extensive hepatic metabolism, as demonstrated by *in vitro* studies using rat and human liver microsomes, which show the production of multiple oxidative metabolites (Zhu et al., 2010). The liver’s metabolic capacity for CAG is significantly higher than that of other major organs, highlighting its central role in CAG biotransformation (Yu et al., 2018). It is worth mentioning that the gut microbiota also plays a certain role in the pharmacokinetics of CAG, as it can convert the precursor AST into CAG, thereby significantly affecting its systemic exposure and therapeutic efficacy (Jin et al., 2015).

The main CAG excretion route is through bile and feces, with the kidneys also participating in partial clearance. Enterohepatic circulation has been observed in animal studies, which may contribute to the prolonged retention time and systemic exposure of CAG (Jin et al., 2015). This recycling effect could enhance the therapeutic window, but could also complicate pharmacokinetic predictions.

Furthermore, CAG has a better therapeutic effect compared to AST, possibly because AST is hydrolyzed into CAG *in vivo*. As the main metabolite of AST after intestinal bacterial biotransformation, CAG has better pharmacological activity than AST. At the same molar concentration (CAG 62.5 mg/kg, equivalent to AST 100 mg/kg), CAG is more effective than AST in inhibiting isoprenaline (ISO) induced cardiac fibrosis and activation of the NLRP3 inflammasome. The enhanced pharmacological action of CAG may be due to the fact that it does not require intestinal bacterial biotransformation, thus avoiding the loss of active saponin aglycone during the transformation process and significantly improving bioavailability (Wan et al., 2018).

### 3.2 Synergistic pharmacokinetics: interactions between cycloastragenol and co-administered agents

**FIGURE 2:**
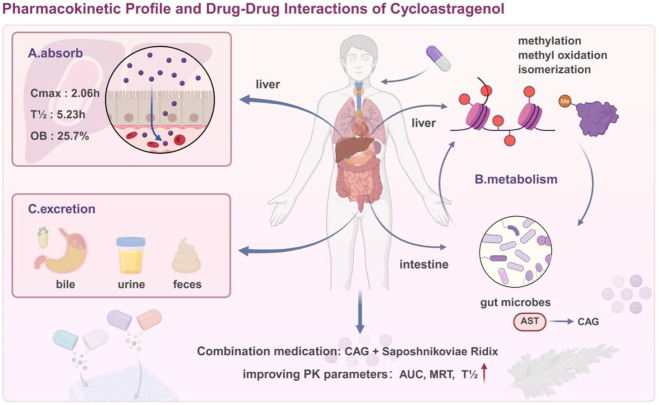
Pharmacokinetic profile and drug-drug interactions of cycloastragenol.

Combination therapy is a common strategy for natural medicines to exert better efficacy. Studying the pharmacokinetic characteristics of drug combinations can help optimize the therapeutic effect of CAG (Figure 2). Currently, research on the interaction between cycloastragenol (CAG) and other drugs focuses mainly on the two dimensions of the metabolic enzyme inhibition effect and the pharmacodynamic synergistic/attenuating effect of the combined drug, and does not involve the typical complex pharmacokinetic interference type. The characteristics and regulation mechanism of its action are described as follows.

#### 3.2.1 Potential herb-drug interactions mediated by UGT enzyme-targeted inhibition

Due to the fact that CAG can inhibit multiple UDP-glucuronosyltransferase (UGT) subtypes, especially UGT1A8 and UGT2B7, IC_50_ values of 0.84 μM and 11.28 μM, respectively. Inhibition was competitive for UGT1A8 and non-competitive for UGT2B7, with Ki values as low as 0.034 μM and 20.98 μM, indicating that elevated systemic exposure to CAG may markedly alter the elimination of co-administered drugs metabolized by these isoforms. This inhibition may alter the metabolism and elimination of both CAG itself and co-administered drugs, so it is possible to predict the likelihood of inhibitory drug interactions (Ran et al., 2016).

From the perspective of clinical application, when the plasma CAG concentration exceeds 0.034 μM (UGT1A8 inhibition threshold) or 20.98 μM (UGT2B7 inhibition threshold), it can delay glucuronidation metabolism of commonly used UGT substrate drugs such as morphine, valproic acid, mycophenolic acid and acetaminophen, resulting in increased exposure of such drugs in the body, and the occurrence of dose-dependent toxic reactions should receive special attention.

#### 3.2.2 Pharmacodynamic synergy: a dual effect of toxicity attenuation and efficacy enhancement

In the combined medication scenario, CAG shows the unique pharmacodynamic characteristics of “reducing toxicity and increasing efficacy,” which provides an important basis for clinical combined medication: CAG has the effect of reducing toxicity. In the study of the gastric cancer model, paclitaxel monotherapy can cause obvious liver and kidney toxicity; after the combined CAG intervention, the biochemical indexes related to liver and kidney function were significantly improved and the tumor inhibition rate did not decrease. Mechanically, CAG relieves the oxidative stress injury induced by paclitaxel, thus achieving the effect of protecting the liver and kidney and reducing toxicity (Wu et al., 2024).

On the other hand, CAG also has the effect of enhancing efficacy. Drug interaction studies have shown that the combined use of CAG and Saposhnikoviae Radix significantly altered the pharmacokinetic parameters of CAG, manifested as increased AUC, prolonged MRT, and prolonged T_1/2_. These results suggest that the combined use of Saposhnikoviae Radix can enhance systemic exposure to CAG, slow its elimination, and prolong its biological activity and potential efficacy (Xiang et al., 2025). Furthermore, in colon cancer cell experiments, the combined use of CAG and 5-fluorouracil (5-FU) or doxorubicin can synergistically enhance activation of the p53-mediated apoptosis pathway, and no additive effect of single-drug toxicity is observed (Park et al., 2022).

The interaction between CAG and other drugs presents a two-way regulation characteristic: on the one hand, it can lead to an increase in the *in vivo* exposure of some combined drugs by strongly inhibiting the metabolic pathway UGT1A8/2B7; on the other hand, it relies on the multi-target pharmacodynamic mechanism of “anti-inflammatory-antioxidant-proapoptotic” to achieve the reduction of toxicity or increase of efficacy of combined drugs. Based on this, personalized strategies should be formulated in combination with the type of substrate drug and the route of administration in clinical combined medication: for UGT metabolism-dependent drugs, the dose should be appropriately adjusted according to the CAG exposure concentration; for treatment regimens that need synergistic efficacy, the combined use ratio can be optimized under the premise of monitoring safety indicators, to maximize therapeutic benefit while ensuring medication safety.

In summary, CAG’s pharmacokinetic and metabolic properties are shaped by various factors, including gut microbiota, hepatic metabolism, and enzyme interactions. While its oral bioavailability presents a notable challenge, advances in drug formulation and combination therapies hold promise to enhance its systemic exposure and therapeutic efficacy. Future research should aim to uncover the precise mechanisms driving the interaction between the gut microbiota and CAG metabolism, design innovative delivery systems to overcome bioavailability limitations, and further evaluate its drug interaction profiles to optimize its clinical use. These studies will provide critical information on unlocking the full therapeutic potential of CAG.

## 4 Molecular mechanisms of CAG in inflammation regulation

Inflammation is a key pathological process in many chronic diseases that involves complex cellular signaling pathways and molecular mechanisms. CAG, a hydrolyzed derivative of AST, has garnered significant attention in recent years for its remarkable anti-inflammatory activity. The core of the anti-inflammatory effect lies in suppressing excessive immune responses and restoring an imbalanced immune microenvironment. Unlike targeting a single pathway independently, its anti-inflammatory effects rely on a core regulatory hub that mitigates oxidative stress, which sequentially drives the coordination of multiple downstream signaling pathways and molecular mechanisms. This integrated regulatory network endows CAG with significant therapeutic potential in a range of inflammation-related diseases. Below, we focus on the detailed and interconnected mechanisms underlying the anti-inflammatory effects of CAG.

### 4.1 Anti-inflammatory mechanisms of cycloastragenol

#### 4.1.1 Modulation of inflammation-related cytokines

Cycloastragalol (CAG) exerts its anti-inflammatory effects by dual-regulating the network of pro-inflammatory and anti-inflammatory cytokines. Its mechanisms involve key signaling pathways and cytokines, and all regulatory actions center on reducing oxidative stress as a core hub. This multi-target regulatory mechanism enables CAG to achieve balance in complex inflammatory environments, effectively alleviating inflammatory responses.

##### 4.1.1.1 Inhibition of pro-inflammatory cytokine secretion

CAG significantly inhibits pro-inflammatory cytokine secretion by modulating multiple inflammation-related signaling pathways. This mechanism mainly involves the signaling systems of nuclear factor κB (NF-κB) and transforming growth factor β (TGF-β), with oxidative stress as the upstream link regulating both pathways.

In the inhibition of pro-inflammatory pathways, CAG primarily targets the two key pathways of NF-κB and TGF-β, which are not regulated independently but are interconnected and work synergistically. NF-κB plays a highly conserved and pivotal role in the inflammatory response. Under resting conditions, NF-κB is sequestered in the cytoplasm through its interaction with the inhibitory protein IκB (Oeckinghaus and Ghosh, 2009). Upon inflammatory stimulation, such as cytokines or damage signals, the NF-κB pathway is activated, resulting in phosphorylation and ubiquitin-dependent degradation of IκBα (Karin, 1999; Oeckinghaus and Ghosh, 2009). This process releases NF-κB, enabling its translocation to the nucleus, where it initiates the transcription of downstream genes such as tumor necrosis factor-α (TNF-α), adhesion molecules, and IL-6 (Shih et al., 2015). Notably, excessive oxidative stress (ROS) is an important trigger for NF-κB pathway activation, CAG targets the upstream oxidative stress hub to reduce ROS accumulation. CAG effectively delays the expression and release of proinflammatory cytokines by suppressing NF-κB nuclear translocation and transcriptional activity, thereby mitigating the spread of inflammation (Li et al., 2020; Xiao et al., 2025). For example, in animal models of Stroke’s disease, CAG treatment induces p65 deacetylation, inhibiting NF-κB activation and thus reducing the expression of pro-inflammatory factors and glial activation (Xiao et al., 2025).

The regulation of the TGF-β signaling pathway by CAG is synergistic with the inhibition of NF-κB. TGF-β, a crucial promoter in chronic inflammation, also exhibits multilayered interactions with NF-κB. In breast cancer models, inhibiting NF-κB signaling significantly eliminates TGF-β-induced epithelial-mesenchymal transition (EMT) induced by TGF-β. TGF-β upregulates the lncRNA NKILA, and the negative feedback mediated by NKILA affects TGF-β-induced NF-κB activation (Wu et al., 2018). By mitigating oxidative stress, CAG reduces the secretion of various pro-inflammatory factors by inhibiting the activity of TGF-β and decreasing its interaction with the NF-κB signaling pathway. Studies have shown that E-CG-01 significantly inhibits the increase in TGF-β expression induced by bleomycin (Kilic et al., 2024).

##### 4.1.1.2 Enhancement of anti-inflammatory cytokine activity

In addition to suppressing pro-inflammatory pathways, CAG further alleviates inflammation by enhancing the activity of anti-inflammatory cytokines and their associated pathways. These anti-inflammatory mechanisms are closely related to the regulation of upstream oxidative stress and the inhibition of the downstream pro-inflammatory pathway, forming a coordinated anti-inflammatory network. Mostly involved the nuclear factor erythroid 2-related factor 2 (Nrf2)/HO-1 signaling pathway and the regulation of IL-10.

CAG mediates anti-inflammatory and antioxidative effects by activating the Nrf2, which is the core pathway for CAG to alleviate oxidative stress and a key link connecting anti-oxidation and anti-inflammation. Under normal conditions, Nrf2 is inactivated through its interaction with the inhibitory protein Keap1. During oxidative stress or inflammation, Keap1 undergoes oxidation, allowing Nrf2 to translocate into the nucleus and initiate the transcription of downstream antioxidant genes, such as HO-1 (Ahmed et al., 2017). HO-1, as an effector enzyme with multifaceted anti-inflammatory functions, produces metabolites (e.g., biliverdin and carbon monoxide) that not only eliminate ROS to further alleviate oxidative stress, but also enhance anti-inflammatory effects by directly inhibiting NF-κB signaling, forming a positive feedback loop between antioxidation and anti-inflammation (Chen et al., 2025b; Thomas et al., 2022). Studies have demonstrated that CAG activates Nrf2, thereby upregulating the expression of HO-1, significantly reduces the release of proinflammatory mediators, and suppresses immune cell infiltration at sites of inflammation (Chen et al., 2022). Moreover, the collective findings indicated that CAG administration is effective in regulating oxidative stress, as shown by reduced expression of LPO and ROS, and improved the expression of Nrf2 and neurogenic processes, modulating MAP kinase-mediated neuroinflammation, apoptotic cell death, and cognitive impairment (Ikram et al., 2021). Furthermore, the degradation products of HO-1 collaborate to neutralize oxidative damage, reduce oxidative stress and cellular injury, and further block pro-inflammatory signaling pathways (Consoli et al., 2021; Ryter, 2021). This mechanism serves as the foundation for CAG’s multi-layered anti-inflammatory regulation.

In addition to the Nrf2/HO-1 pathway, CAG can also enhance the anti-inflammatory effect by regulating the key anti-inflammatory cytokine IL-10. IL-10, one of the most critical anti-inflammatory cytokines, reduces the intensity of proinflammatory signals by directly inhibiting proinflammatory cytokines (e.g., TNF-α, IFN-γ, and IL-17) and suppressing the activation of the signaling pathways NF-κB and JAK-STAT (Carlini et al., 2023). By mitigating oxidative stress, CAG induces M2 macrophage polarization, promoting IL-10 secretion to enhance anti-inflammatory activity (Chen et al., 2022). For example, in certain experimental models, CAG modulates the balance of T helper (Th) cells by suppressing Th1/Th17 cell activity (pro-inflammatory T cells) while enhancing the expression of Th2-associated cytokines such as IL-4 and IL-10, thereby systemically controlling systemic inflammatory responses (Ren et al., 2020; Sun et al., 2017). This unique feature underscores its therapeutic potential in various models of inflammatory diseases.

This section clarifies that CAG exerts anti-inflammatory effects through dual regulation of the pro-inflammatory/anti-inflammatory cytokine network, with the core mechanism centered on alleviating oxidative stress, involving the inhibition of NF-κB nuclear translocation and the activation of the Nrf2/HO-1 pathway. Existing evidence shows that CAG can dose-dependently reduce the levels of pro-inflammatory factors such as TNF-α and IL-6, while promoting the release of anti-inflammatory mediators such as IL-10 (Chen et al., 2022). This bidirectional regulatory characteristic distinguishes it from traditional anti-inflammatory drugs (such as NSAIDs that only inhibit COX-2) and provides new ideas for the treatment of chronic inflammatory diseases. CAG’s regulation of Th1/Th17/Th2 cell balance suggests its potential application value in autoimmune diseases (Sun et al., 2017), but spatiotemporal-specific regulation of the cytokine profile still needs further research.

#### 4.1.2 Suppression of the NLRP3 inflammasome

The NLRP3 inflammasome is a crucial multi-protein complex in the innate immune system, composed of the pattern recognition receptor NLRP3, the adaptor protein ASC and the effector protease caspase-1 (Deng et al., 2019). NLRP3 can detect a variety of pathogen-associated molecular patterns (PAMPs) and damage-associated molecular patterns (DAMPs). ASC, through its PYD domain, connects NLRP3 to caspase-1, while caspase-1 is responsible for mediating the cleavage and maturation of interleukin-1β (IL-1β) and IL-18 precursors and triggering pyroptosis (Blevins et al., 2022). Pyroptosis is a programmed cell death pathway mediated by Gasdermin family proteins, characterized by perforation of the cell membrane, cell swelling and rupture, and the release of large amounts of pro-inflammatory factors. Unlike apoptosis, pyroptosis triggers a strong inflammatory response, which is protective against intracellular pathogen infection, but overactivation can also lead to pathological damage such as sepsis, autoimmune diseases, and neurodegenerative diseases (Broz, 2025).

In particular, the above inhibitory effects of CAG are closely related to its upstream regulation: on the one hand, CAG alleviates oxidative stress to reduce ROS-induced activation of the NLRP3 inflammasome; on the other hand, CAG can inhibit activation of the NLRP3 inflammasome through several complementary mechanisms. *In vitro* studies using bone marrow-derived macrophages stimulated by lipopolysaccharide (LPS), CAG significantly inhibited the expression of the caspase-1 p20 fragment, indicating reduced enzyme activity. This inhibition directly suppresses the release of IL-1β and IL-18, thus attenuating inflammatory signaling. CAG can also disrupt the assembly of the NLRP3 inflammasome complex by inhibiting the interaction between ASC and NLRP3, further preventing the recruitment and activation of caspase-1, effectively blocking downstream inflammatory processes. Meanwhile, *in vitro* experiments using imiquimod-stimulated macrophages (IMQ) further confirmed that CAG inhibits NLRP3 inflammasome assembly and downstream cytokine production (Deng et al., 2019). In the cardiac fibroblast model, CAG significantly inhibits the overexpression of NLRP3, caspase-1, IL-18, and IL-6 mRNA, suggesting that it alleviates excessive collagen synthesis in cardiac fibroblasts by inhibiting the NLRP3 inflammasome pathway (Wan et al., 2018). Studies have found that CAG specifically regulates NLRP3 inflammation-related proteins in primary microglia: CAG reduces the expression of NLRP3, caspase-1 and cleaved GSDMD in primary microglia induced by α-Syn, thus effectively inhibiting activation of the NLRP3 inflammasome and pyroptosis in primary microglia induced by α-Syn (Feng et al., 2024).

Furthermore, the anti-inflammatory effects of CAG by inhibiting the NLRP3 inflammasome have also been validated *in vivo* models. In the mouse model with IMQ-induced psoriasis, a disease characterized by inflammasome-driven skin inflammation. After CAG intervention, inflammation was significantly reduced and symptoms similar to psoriasis were alleviated. Histological analysis showed that CAG administration reduced caspase-1 activation and IL-1β secretion in skin tissue. The results indicate that CAG alleviates inflammation by inhibiting caspase-1-mediated pyroptosis and inflammatory cytokine release (Deng et al., 2019).

This section systematically demonstrates the molecular mechanism by which CAG blocks the assembly and activation of the NLRP3 inflammasome, which is regulated by upstream CAG oxidative stress alleviation and activation of the AMPK pathway. Experimental data show that CAG significantly reduces the generation of caspase-1 p20 fragments (Deng et al., 2019) and inhibits the interaction between ASC and NLRP3, an effect that has been verified in psoriasis (IMQ model) and neuroinflammation (α-Syn model) (Feng et al., 2024). The significance of this mechanism lies in the fact that the overactivation of NLRP3 is closely related to many chronic diseases (such as diabetes and Alzheimer’s disease), and CAG’s targeted inhibition may avoid the systemic side effects of glucocorticoids. However, it is not clear whether CAG affects other inflammasomes (such as AIM2 or NLRC4), and comparative studies will need to be carried out in the future.

#### 4.1.3 Regulation of inflammatory signaling pathways

CAG exerts effective anti-inflammatory effects by modulating key signaling pathways, primarily by activating AMP-activated protein kinase (AMPK) and regulating the activity of mitogen-activated protein kinase p38 (p38-MAPK). These pathways are crucial for cellular metabolism and inflammation regulation, and also interact with the core oxidative stress hub and other downstream pathways, forming a complex regulatory network and playing a key role in attenuating inflammatory responses under pathological conditions. The AMPK pathway acts as a central regulatory hub for the metabolism of cellular energy. When cells are under energy stress, AMPK is activated by an increased AMP/ATP ratio (Steinberg and Hardie, 2022), which inhibits the NF-κB pathway by directly phosphorylating the p65 subunit (Salminen et al., 2011), reducing excessive inflammation (Rodríguez et al., 2021).

In addition, the AMPK pathway is closely related to the autophagy process. Activated AMPK promotes autophagy by inhibiting mTORC1 (Salt et al., 2024), a process that can clear damaged organelles and misfolded proteins, thereby alleviating inflammatory responses triggered by endoplasmic reticulum stress and mitochondrial dysfunction (Su and Dai, 2017; Wang et al., 2016). Mitochondrial dysfunction is an important source of ROS, so AMPK-induced autophagy can also synergize with the Nrf2/HO-1 pathway to further alleviate oxidative stress. In non-small cell lung cancer (NSCLC) cells, CAG activates AMPK/ULK1/mTOR to promote autophagy, inhibit inflammatory signaling, and exert anti-tumor effects (Zhu et al., 2024). Furthermore, CAG activates AMPK to inhibit the thioredoxin-interacting protein (TXNIP), a key mediator of inflammation and oxidative stress that can directly activate the NLRP3 inflammasome. CAG promotes the degradation of TXNIP *via* AMPK to reduce TXNIP expression. Studies have shown that palmitic acid (PA)-induced inhibition of AMPK leads to increased levels of TXNIP and activation of NLRP3, both of which are reversed after CAG treatment. The use of AMPK inhibitors (e.g., Compound C) significantly reduces the impact of CAG’s on TXNIP, confirming the central role of AMPK in mediating its anti-inflammatory results (Zhao et al., 2015).

Beyond its effects on AMPK, CAG also affects the MAPK pathway, which has a dual role in inflammation: it can act as a positive regulator to promote inflammation and as a negative regulator to inhibit inflammation. The MAPK family, particularly p38-MAPK, plays a role in regulating the synthesis of inflammatory mediators, making it a potential target for anti-inflammatory therapy (Kaminska, 2005). On the one hand, activation of p38-MAPK exacerbates inflammation by promoting ASC phosphorylation and assembly of the NLRP3 inflammasome (Dong et al., 2021). On the other hand, p38 MAPK also exhibits anti-inflammatory effects and plays a key role in the regulation of the biosynthesis of pro-inflammatory cytokines such as IL-1β and TNF-α (Machado et al., 2021). CAG regulates p38-MAPK to inhibit inflammation, and this regulation is closely linked to oxidative stress: in LPS-induced inflammatory models, CAG inhibits MAPK to suppress local arterial inflammation (Chen et al., 2025a). In models of abdominal aortic aneurysm (AAA), CAG can inhibit inflammation, oxidative stress, and the expression and activity of MMPs by blocking the MAPK signaling pathway. But the inhibition of inflammation and oxidation facilitates the inactivation of the MAPK signaling pathway (Wang et al., 2018b). This dual function highlights the value of p38-MAPK as a tunable target in the management of inflammation. Similarly, in Aβ-injected mice, CAG upregulates Nrf2 *via* mitigating oxidative stress to restore MAPK expression and inhibit p-JNK, alleviating neurodegenerative inflammation (Ikram et al., 2021).

Regulation of the AMPK/p38 MAPK signaling axis by CAG reveals the characteristics of its metabolic-inflammatory crosstalk. Studies have shown that CAG inhibits the TXNIP/NLR3 pathway by activating AMPK (Zhao et al., 2015), while reducing IL-1β secretion by downregulating phosphorylation of p38 MAPK (Wang et al., 2018a). This multi-target property has shown a synergistic effect in models of ischemic brain injury and lung cancer. It is particularly noteworthy that the AMPK/ULK1/mTOR-dependent autophagy induced by CAG provides a new perspective for explaining its cytoprotective effects (Zhu et al., 2024). However, the dual role of p38 MAPK in inflammation (pro-inflammatory/anti-inflammatory) suggests that tissue-specific effects should be guarded against, and future studies should be combined with conditional gene knockout models for verification.

#### 4.1.4 Mitigating oxidative stress *via* cycloastragenol

Oxidative stress, the imbalance between reactive ROS production and antioxidant defenses, is a key driver of inflammation (Sadasivam et al., 2022). Excess ROS leads to neutrophil infiltration, protease release, and activation of inflammatory pathways, which, in turn, triggers excessive production of inflammatory mediators (Mittal et al., 2013). As the core upstream hub of CAG’s anti-inflammatory mechanisms, mitigating oxidative stress is the foundation for all subsequent regulatory effects. CAG alleviates oxidative stress by activating the Nrf2/ARE signaling pathway, enhancing antioxidant enzyme activity, and maintaining mitochondrial function, further reducing inflammation (Yilmaz et al., 2022). In microglia, CAG suppresses ROS-induced NLRP3 inflammasome activation by enhancing autophagy and inhibiting the Scrib/p22phox complex, reducing the release of inflammatory cytokines (Feng et al., 2024). In an ischemic brain injury model, CAG protects nerve tissue by inhibiting mitochondrial damage and NF-κB-mediated inflammatory responses, reducing TNF-α and IL-1β. These results suggest that CAG has the potential to mitigate mitochondrial-derived oxidative stress and related inflammation (Li et al., 2020).

Nrf2 is a crucial transcription factor that coordinates cellular responses to oxidative stress. Under normal conditions, Nrf2 binds to Keap1 and is inactivated in the cytoplasm. Under conditions of oxidative stress, Keap1 undergoes oxidative modification, releasing Nrf2, which then translocates to the nucleus and forms a heterodimer with small Maf proteins. These complex bind to ARE, initiating the transcription of antioxidant genes (Kanzaki et al., 2013). Studies have shown that CAG significantly enhances cellular antioxidant capacity and reduces oxidative stress-induced cell damage and inflammation by modulating the Nrf2/ARE pathway. CAG promotes Nrf2 nuclear translocation, activates ARE, and upregulates the expression of antioxidant enzymes such as HO-1, GR and GCLC, thereby scavenging excess ROS and reducing oxidative damage (Yilmaz et al., 2022). Furthermore, the study found that under RANKL stimulation, Keap1 undergoes cleavage and dissociates from Nrf2, allowing Nrf2 to escape and enter the nucleus. CAG treatment can enhance the transcriptional activity of ARE blocked by RANKL, upregulate the expression of Nrf2, the Nrf2/Keap1 ratio, and the induction of antioxidant enzymes observed in RTqPCR. Regulation of the RANKL-induced Nrf2/Keap1/ARE pathway may play a potential role in the inhibitory effect of CAG on RANKL-induced osteoclastogenesis (Wang et al., 2022b).

**FIGURE 3:**
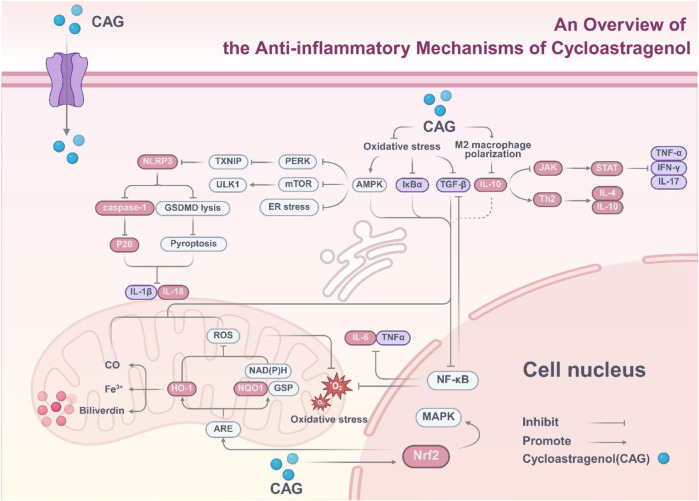
Overview of the anti-inflammatory molecular mechanisms of cycloastragenol polymorphisms.

In summary, CAG is a natural compound with significant anti-inflammatory effects, exerting its regulatory role through an integrated network centered on mitigating oxidative stress (core upstream hub). This hub sequentially drives five interconnected mechanisms: (1) inhibiting pro-inflammatory cytokine secretion *via* suppressing the NF-κB/TGF-β pathway; (2) enhancing anti-inflammatory cytokine activity *via* activating the Nrf2/HO-1 pathway and promoting IL-10 secretion; (3) inhibiting assembly and activation of NLRP3 inflammasomes; (4) regulating the AMPK/p38-MAPK signaling axis to coordinate metabolic and inflammatory responses; (5) reinforcing the mitigation of oxidative stress through the Nrf2/ARE pathway (Figure 3). In particular, these mechanisms are not independent—for example, Nrf2/HO-1 activation not only eliminates ROS but also inhibits NF-κB and NLRP3; AMPK activation suppresses both TXNIP and NLRP3—forming a synergistic anti-inflammatory network.

Despite its broad anti-inflammatory potential, several questions regarding CAG still need further investigation and resolution. For example, its mechanism of action involves multiple signaling pathways and molecular targets, which increases the complexity of fully understanding its effects. More research is needed to elucidate its specific mechanisms in different inflammatory diseases. In terms of clinical application, more studies are needed to address key issues such as mechanistic complexity and long-term safety to achieve its widespread use in clinical treatment.

### 4.2 Other pharmacological mechanisms of cycloastragenol

CAG, as a natural medicine, exhibits a wide range of pharmacological activities. In addition to its anti-inflammatory effects, CAG also has the ability to inhibit apoptosis triggered by inflammatory responses. When inflammation occurs, it can protect cells from damage caused by inflammation by blocking the process of programmed cell death. In addition, CAG also has important immunomodulatory effects. This means that it can influence and adjust the function of the immune system to make it work better and maintain balance and stability within the body.

#### 4.2.1 Regulation of apoptosis

CAG alleviates inflammation and oxidative stress-induced apoptosis through multi-target synergistic effects, and its protective mechanism exhibits significant network characteristics. In an inflammatory environment, CAG not only directly inhibits the overactivation induced by TNF-α-induced overactivation of the Nrf2/HO-1 pathway (Wang et al., 2018b), but more importantly, this regulation forms a dual protective effect: on the one hand, it reduces pro-inflammatory factor levels to alleviate the inflammatory response, and on the other hand, the activated Nrf2/HO-1 pathway can simultaneously eliminate excess ROS within cells (Ikram et al., 2021). Furthermore, this multi-target characteristic is particularly prominent in the regulation of the MAPK signaling pathway. CAG’s inhibition of JNK1/2 and ERK1/2 phosphorylation (Wang et al., 2018b) actually blocks a key regulatory node of the pro-apoptotic protein Bim, reducing both JNK1/2-mediated Bim-Bax binding (Buder-Hoffmann et al., 2009). and interfering with ERK1/2-dependent Bim degradation (Luciano et al., 2003). This “dual-pronged” effect comprehensively blocks the initiation of cell apoptosis. Notably, CAG’s protective effect is also reflected in the maintenance of cell phenotypic stability. By upregulating the VSMC marker SM22α, it not only maintains cell contraction function, but also constructs a complete anti-apoptotic network from membrane receptors to intranuclear genes by regulating the balance of Bcl-2/Bax (Ikram et al., 2021). This network regulation also extends to the extracellular matrix. By inhibiting MMP activity and promoting fibrillin expression, CAG achieves comprehensive protection from intracellular signals to the extracellular microenvironment, ultimately maintaining the integrity and functionality of vascular structures.

As a self-defense mechanism during cellular stress, certain autophagy signaling pathways can trigger apoptosis. In lung cancer models, CAG induces protective autophagy by regulating the AMPK/ULK1/mTOR pathway. CAG induces the accumulation of LC3 (a traditional autophagy marker) in H1299 and A549 cells. Chloroquine (CQ), an autophagy inhibitor, suppresses CAG-induced autophagy, thereby leading to increased cell apoptosis and accumulation of cleaved PARP, further confirming the enhancement of apoptosis through autophagy blockade. Experiments have shown that inhibiting AMPK expression using siAMPK or Compound C enhances apoptosis in CAG-treated lung cancer cells while also inhibiting cancer cell proliferation. Similarly, under stress conditions, inhibiting ULK1 expression using siULK1 significantly increases cell apoptosis (Zhu et al., 2024).

#### 4.2.2 Immunomodulatory effects of cycloastragenol

CAG exhibits significant immunomodulatory capabilities that involve the regulation of various molecular and immune pathways. For example, CAG inhibits the activity of the target protein cathepsin B, blocking the degradation of class I major histocompatibility complex (MHC-I) in lysosomes. This action promotes the accumulation of MHC-I molecules on the cell membrane, thereby enhancing the presentation of antigens on the surface of tumor cells. As a result, the immune system’s ability to recognize tumor cells is improved (Deng et al., 2022).

Additionally, CAG suppresses T-cell activation and proliferation induced by concanavalin A (Con A). Specifically, it significantly reduces the expression of activation markers, such as CD69 and CD25, on the surface of Con A-activated CD3^+^ T cells and inhibits the proliferation of activated lymphocytes. Further studies reveal that CAG blocks Con A-induced mitotic activity, causing activated lymphocytes to arrest in the G0/G1 phase of the cell cycle, while markedly decreasing the proportion of cells in the S and G2/M phases. These findings suggest that CAG inhibits lymphocyte activation and proliferation by regulating the cell cycle. At the cytokine level, CAG shows prominent regulatory effects on Th1, Th2 and Th17 cytokines in Con A-activated lymphocytes. It suppresses the release of multiple cytokines, including Th1-type cytokines (IFN-γ, TNF, IL-2), Th2-type cytokines (IL-4, IL-6, IL-10) and Th17-type cytokines (IL-17A). This reflects CAG’s comprehensive capacity to modulate both inflammatory and pro-inflammatory immune responses (Sun et al., 2017).

Furthermore, CAG plays a vital role in improving the functionality of CD8^+^ T cells. Research shows that CAG promotes the activation of CD8^+^ T cells, increasing the expression levels of activation markers such as CD69 and CD28 on their surface. It also increases the secretion of key effector molecules, including IFN-γ and granzyme B (GZMB), allowing CD8^+^ T cells to recognize and kill tumor cells more effectively. In addition, CAG inhibits the PD-1/PD-L1 immune checkpoint signaling pathway, effectively reducing CD8^+^ T cell exhaustion and further strengthening their antitumor immune activity (Deng et al., 2022).

In summary, CAG, a versatile natural compound, shows significant effects in reducing inflammation-mediated apoptosis and modulating immune functions. It alleviates oxidative stress and ROS accumulation, inhibits apoptosis, and induces protective autophagy. In addition, it improves antigen presentation, regulates the cell cycle, inhibits lymphocyte activation, suppresses cytokine release, increases anti-tumor immunity, and reduces CD8^+^ T cell exhaustion. Despite its diverse activities in experiments, its clinical application is limited because of an incomplete understanding of its specific mechanisms. More research is needed to clarify its precise actions in various cell types and disease models.

### 4.3 Advantages of cycloastragenol compared to existing anti-inflammatory drugs

Compared with frontline anti-inflammatory drugs in clinical practice (such as nonsteroidal anti-inflammatory drugs, glucocorticoids, JAK inhibitors, *etc.*), CAG, as an active component derived from natural medicinal plants, demonstrates significant multidimensional and highly selective advantages, specifically reflected in the following key aspects:

First, CAG has a narrow spectrum of side effects and they are mild. For example, long-term use of glucocorticoids can induce central obesity, osteoporosis, hyperglycemia, and suppression of the hypothalamic-pituitary-adrenal axis (Jang et al., 2024), while in a 13-week rat toxicology study of CAG (≤150 mg kg-1 day-1), no abnormalities in body weight, bone density, or blood glucose were observed (Szabo, 2014). Nonsteroidal anti-inflammatory drugs (NSAIDs) work by inhibiting COX-1/2. Inhibition of COX-1 leads to a deficiency of gastric mucosal prostaglandins, which can result in gastric erosions and bleeding (Wallace et al., 2000); CAG has minimal effect on COX-1 activity (IC_50_ > 100 μM) and did not cause gastric mucosal damage or bleeding in rodent models. Although selective COX-2 inhibitors reduce gastrointestinal risks, they may increase thrombotic and cardiovascular events (Kearney et al., 2006); CAG, without affecting platelet COX-1, inhibits COX-2 transcription by blocking the NF-κB/NLRP3 axis, theoretically avoiding cardiovascular side effects (Wang et al., 2022a). However, CAG is extracted from natural plants, which makes it natural in origin and low in immunotoxicity. Current studies show that long-term use does not cause these side effects; existing clinical supplements TA-65, taken orally for 12 consecutive months at 32 mg per day, have not shown abnormalities in liver enzymes, kidney function, or blood tests (Salvador et al., 2016).

Compared to biologics, CAG does not induce systemic immunosuppression. The immunosuppressive effect of CAG does not entail a broad downregulation of immune function; rather, it is targeted and context-dependent, with the primary action being the suppression of excessive immune responses and the restoration of an imbalanced immune microenvironment. For example, in an OVA-induced asthma model, CAG inhibits airway epithelial autophagy by downregulating LC3B/Beclin-1 and upregulating p62 in lung tissue, reduces the presentation of MHC-II antIgEns, lowers levels of IL-5, IL-13, and IgE, and decreases eosinophil infiltration. Notably, this effect is specific to Th2-dominant inflammation and does not significantly suppress Th1 or CTL responses (Zhu et al., 2021). In the tumor context, direct targeting of the A77/G198 sites of tissue protease B (CTSB) can block the lysosomal pathway of tumor cells responsible for MHC-I degradation, thus increasing the surface abundance of MHC-I to enhance the recognition and cytotoxicity of CD8^+^ T cells. This mechanism is only evident in tumor microenvironments with high CTSB expression and low MHC-I expression (Deng et al., 2022). It can also use ROS levels as a threshold switch to reduce IL-4/IL-5/IL-13 transcription by suppressing JAK2/STAT6 phosphorylation and downregulating GATA-3 (Zhu et al., 2022), while simultaneously blocking NF-κB p65 nuclear translocation to decrease the release of pro-inflammatory factors such as TNF-α and IL-6 (Chen et al., 2025a; Li et al., 2020). This mechanism is only activated when oxidative stress exceeds a critical threshold and has no effect on immune cell cytokine secretion under homeostatic conditions (Ikram et al., 2021). In summary, the immunosuppressive effects of CAG exhibit context-, target-, and cell type-specific selectivity. Relevant pathological contexts include high Th2 expression, improved autophagy, or elevated CTSB activity. Its targets encompass LC3B/Beclin-1, CTSB, and others, while the affected cell types are primarily overactivated Th2 cells and eosinophils. CAG functions as an ‘immune calibrator’ rather than a conventional immunosuppressant, providing a precise immune reprogramming strategy for use in anti-allergy, anti-asthma, and combined tumor immunotherapy.

Finally, CAG has the unique advantage of multi-target synergistic regulation, which significantly differentiates it from traditional single-path-targeted anti-inflammatory drugs. Most clinically used anti-inflammatory drugs currently focus on a single signaling pathway or molecular target: non-steroidal anti-inflammatory drugs (NSAIDs) exert their effect primarily by selectively inhibiting the activity of cyclooxygenase-2 (COX-2), thus blocking the synthesis of prostaglandin inflammatory mediators, and only targeting the downstream effector stages of the inflammatory cascade (Bosch et al., 2022). Glucocorticoids rely on binding to the glucocorticoid receptor (GR) and regulate the transcription of genes related to inflammation by activating the GR-GRE (glucocorticoid response element) signaling pathway. Although they have a broad range of effects, they lack precise targeting (Kadmiel and Cidlowski, 2013). JAK inhibitors function primarily by selectively blocking the kinase activity of the JAK family (such as JAK1/2/3), suppressing the activation of downstream STAT signaling pathways, and acting only on the cytokine-mediated segments of inflammatory signal transduction. They lack integrated regulation of oxidative stress and the immune microenvironment, making it difficult to address the multi-pathway, overlapping pathological features of chronic inflammation (Mathew et al., 2024; Charras et al., 2019). In contrast, CAG, with the regulation of oxidative stress as its central upstream hub, has established a synergistic anti-inflammatory model that encompasses inhibition of pro-inflammatory pathways, activation of anti-inflammatory pathways, and regulation of inflammasomes, making it more suitable for the complex pathological characteristics of chronic inflammation. From a molecular mechanism perspective, CAG can simultaneously and precisely regulate multiple core inflammation-related pathways and functional networks: On one hand, by inhibiting NF-κB p65 nuclear translocation and blocking the assembly and activation of the NLRP3 inflammasome, it directly suppresses the release of pro-inflammatory factors such as TNF-α, IL-6, and IL-1β (Deng et al., 2019; Feng et al., 2024); on the other hand, by activating the Nrf2/HO-1 antioxidant pathway, it eliminates excessive reactive oxygen species (ROS) and restores cellular redox balance, thereby mitigating the oxidative stress that underlies inflammation initiation (Yilmaz et al., 2022); simultaneously, it regulates autophagy-related signaling networks and immune cell functional phenotypes (Sun et al., 2017), achieving a triple synergistic effect of anti-inflammation, antioxidant activity, and immune modulation. This multi-target integrated regulatory approach not only allows for synchronous intervention at multiple critical points in the onset and progression of inflammation but also effectively avoids issues such as drug resistance and compensatory inflammatory rebound that are common with single-target therapies—preventing the scenario where blocking a single pathway leads the body to activate other inflammatory pathways as compensation, resulting in suboptimal inflammation control or recurrent disease. It provides a more comprehensive and stable therapeutic strategy for the long-term management of complex chronic inflammatory conditions, such as rheumatoid arthritis, chronic obstructive pulmonary disease, and inflammatory bowel disease.

In summary, compared to first-line clinical anti-inflammatory drugs such as nonsteroidal anti-inflammatory drugs, glucocorticoids, and JAK inhibitors, CAG offers the triple advantages of safety with low toxicity, targeted immune modulation, and multi-target synergistic regulation, establishing a treatment model better suited to the complex pathology of chronic inflammation. Its narrow and mild side effect profile avoids the risks of serious adverse reactions associated with traditional drugs, while its precise inflammatory targeting prevents the risks of infection caused by systemic immunosuppression. At the same time, its multi-pathway integrated intervention focused on oxidative stress regulation overcomes the limitations of drug resistance and compensatory rebound seen with single-target drugs. This series of advantages not only highlights CAG’s unique value in anti-inflammatory therapy but also provides a new direction for long-term safe intervention in chronic inflammation that is both scientific and practical, laying an important foundation for its translation from basic research to clinical application.

## 5 Cycloastragenol: research progress from anti-inflammatory effects to multifield clinical applications

After a basic overview of the anti-inflammatory mechanisms and related biological characteristics of CAG. This section focuses on its specific roles and the latest progress in the treatment of various inflammation-related diseases, with the aim of exploring its clinical potential and prospects. Detailed information on the pharmacological activities and mechanisms of CAG in different disease models is presented in Table 2.

**TABLE 2:** Key information on the pharmacological activities and mechanisms of cycloastragenol.

Disease	Model	Dose	Administration route	Detailed mechanism	References
Cancer	Male Sprague-Dawley rats	125 mg/kg	In vivo	Reduced MMP-2 activity, preserve elastin, and decreased calcification	Melin et al. (2022)
	Adult male C57BL/6 mice	20 mg/kg	In vivo	Inhibition of the activation of the NF-κB and Nrf2 signaling pathways	Chen et al. (2022)
	Male Sprague-Dawley rats	2.5, 5 and 10 mg/kg	In vivo	Inhibition of the MAPK/Nrf2/HO-1 signaling pathway in neurons and astrocytes, suppression of heme-induced apoptosis, and promotion of proliferation in HT22 cells.	Ying et al. (2022)
	male C57BL/6 mice	125 mg·kg^−1^	In vivo	Downregulation of the MAPK signaling pathway, improvement of the expression and activity of MMPs, reduction of the activation of the ERK/JNK signaling pathway, and inhibition of elastin degradation.	Wang et al. (2018b)
Neuroinflammation	Male C57BL/6 mice	5, 10 or 20 mg/kg	In vivo	Upregulation of SIRT1 expression, deacetylation of p53, inhibition of NF-κB activation, and suppression of cell apoptosis and neuroinflammation.	Li et al. (2020)
	BV2	1, 5 or 10 μM	In vitro	CAG rather than AST inhibits the proliferation and migration of microglia *in vitro*.	Hu et al. (2023)
	C57BL/6 N mice	20 mg/kg	In vivo	Inhibition of oxidative stress, neurotrophic factors, MAP kinases, and apoptosis-related markers	Ikram et al. (2021)
	SH-SY5Y cells C57BL/6 mice	5, 10, 20, 40, 80, 100 μM 10 mg/kg, 30 mg/kg	In vitro	Targeted and activated the expression of Fpr2, inhibited the TLR4/NF-κB signal pathway, enhanced neuronal viability, and suppressed the expression of inflammatory factors.	(Xiao et al., 2025)
	Male C57BL/6 mice	75 mg/kg, 125 mg/kg	In vivo	Downregulation of Scrib, enhance autophagy, mitochondrial protection, reduced oxidative stress, and inhibition of NLRP3 inflammasome activation	(Feng et al., 2024)
	adult male Sprague-Dawley rats	5 mg/kg,10 mg/kg, 20 mg/kg	In vivo	Promoted the expression of SIRT1 and FoxO1, downregulated NF-κB activity, and promoted the deacetylation of p53.	(Lin et al., 2022)
Asthma	BALB/c female mice	125 mg·kg^−1^	In vivo	Inhibited the expression of p38 MAPK, the levels of ITGAL, Syk, and reduced Vav1, thereby decreased the production of cytokines.	(Zhu et al., 2022)
	BALB/c female mice	31.25 mg/kg,62.5 mg/kg,125 mg/kg	In vivo	Exhibited anti-inflammatory effects and inhibited autophagy, as evidenced by decreased LC3B protein expression and increased p62 protein expression.	(Zhu et al., 2021)
Anti-visceral fibrosis	male BALB/c mice	31.25 mg/kg/d or 62.5 mg/kg/d	In vivo	Inhibition of the NLRP3 inflammasome pathway, downregulation of TGF-β1 expression, and reduction in the mRNA expression of collagen-1 and collagen-3.	(Wan et al., 2018)
	Wild-type C57BL/6 mice	2 mg/kg/day, 6 mg/kg/day, 10 mg/kg/day	In vivo	Reduced ROS levels, enhance antioxidant enzymes, and decreased collagen deposition, TGF-β expression, and Ki-67 expression.	(Kilic et al., 2024)
	Male ICR outbred mice	50 mg/kg, 200 mg/kg	In vivo	Upregulation of anti-fibrotic MMPS and inhibition of the non-canonical TGF-β1/Akt signaling pathway	(Luangmonkong et al., 2023)
	WT, NLRP3 KO, and GSDMD KO mice	125 mg/kg	In vivo	Inhibition of NLRP3 inflammasome-mediated pyroptosis and inhibition of the TGF-β/Smads signaling pathway	Qi (2024)
Other inflammatory diseases	EA. hy926 cells	10 μM	In vitro	Inhibition of ER stress-associated oxidative stress, inhibition of the TXNIP/NLRP3 inflammasome, and inhibition of pro-inflammatory cytokines.	(Zhao et al., 2015)
	Sprague Dawley rats	30 mg/kg	In vivo	Blocked the UC-induced increased in SphK, MIP-1α, and NF-κB expression, as well as the decreased in miR-143 expression	(Bagalagel et al., 2022)
	Female C57BL/6 mice	12.5,25,50 mg/kg	In vivo	Inhibition of NLRP3 inflammasome-mediated pyroptosis of macrophages and suppression of mRNA expression of IL-17a, TNF-α, and IL-1β	(Deng et al., 2019)
	MLE-12 cell	0.01,0.1 and 1 μM	In vitro		Zhu et al. (2023)
Lipid metabolism	HepG2 (ATCC) cells	25 μM	In vitro	Stimulated FXR transcriptional activity, reduced fasting plasma glucose and triglyceride levels, decreased hepatic TBA levels, and induced the mRNA expression of hepatic Nr0b2.	(Gu et al., 2017)
	Female C57BL/6	100 mg/100 g diet	In vivo		
	adult male C57BL/6 J (wild-type AQP9) mice	100 g kg-1mg kg-1/d	In vivo	Upregulated the expression of AQP9 in hepatocytes, reduced ROS, H_2_O_2_ and malondialdehyde, and activated the PI3K-AKT and insulin signaling pathways.	Li et al. (2024)
Bone protection	MC3T3-E1 cells	0.03–3 μM	In vitro	The expression of osteoclastogenic factors, TRACP levels, malondialdehyde levels, and the number of osteoclasts were reduced.	Yu et al. (2020)
	C57BL/6J mice	0, 1, 2.5, 5, 7.5, 10 μM	In vitro	Enhanced the Nrf2/Keap1/ARE pathway and inhibited the NF-κB and calcium pathways, as well as downregulated NFATc1 and c-Fos.	Wang et al. (2022b)
	female Sprague–Dawley (SD) rats	5 and 15 mg/kg	In vivo	Inhibited hyperactive osteoclasts, reduced the gene and protein expression of CTSK, and alleviated blood supply.	Wang et al. (2024a)

### 5.1 Cycloastragenol: a new strategy for disease intervention by targeting inflammatory pathways

#### 5.1.1 Cycloastragenol in cancer therapy: targeting inflammation and tumor microenvironment

The anti-inflammatory properties of CAG have been preliminarily demonstrated in numerous studies, offering new intervention strategies for the treatment of inflammation-related diseases. In the AAA model, CAG significantly reduces inflammatory responses by inhibiting the MAPK signaling pathway (particularly the phosphorylation of ERK and JNK). This mechanism not only blocks the macrophage-mediated inflammatory cascade but also directly protects the function of vascular smooth muscle cells (VSMC). Specifically, CAG inhibits TNF-α-induced MMP induced by TNF-α on the one hand, maintaining the stability of the extracellular matrix (ECM); on the other hand, it promotes the expression of fibrillin-1 and fibulin-5, further consolidating the structural integrity of the vascular wall. Furthermore, CAG reduces ROS accumulation by upregulating the Nrf2/HO-1 antioxidant pathway, while downregulating pro-inflammatory factors (such as MCP-1 and IL-6), forming an anti-inflammatory-antioxidant synergy to comprehensively alleviate vascular inflammation and damage from oxidative stress (Wang et al., 2018b).

CAG’s anti-inflammatory mechanism also shows significant therapeutic potential in the tumor microenvironment. Unlike traditional non-selective COX inhibitors, CAG can precisely inhibit the NF-κB/COX-2/PGE2 inflammatory axis of NF- κB/COX-2/PGE2, reducing the release of prostaglandin E2 (PGE2), thus inhibiting tumor-related inflammation and proliferation, while avoiding gastrointestinal side effects caused by inhibition of COX-1. Moreover, CAG can regulate the tumor immune microenvironment, for example, by inhibiting TAM (tumor-associated macrophage)-mediated hypoxia signaling and inflammatory responses, and regulating lymphocyte activation and cytokine expression, thereby limiting tumor growth (Debeleç-Bütüner et al., 2018).

More strikingly, CAG’s anti-inflammatory effect is closely related to its anti-apoptotic mechanism, giving it dual benefits in cancer treatment. For example, in cervical cancer, CAG reverses abnormal proliferation and apoptosis resistance of tumor cells by upregulating the expression of PIF1 and telomerase reverse transcriptase (TERT), providing new molecular targets for cancer treatment (Wang et al., 2020). These findings collectively indicate that the anti-inflammatory effect of CAG’s is not isolated but forms a networked therapeutic effect by regulating multiple aspects, including inflammation, oxidative stress, cell apoptosis, and the immune microenvironment, offering a more comprehensive strategy for the intervention of complex inflammatory diseases.

#### 5.1.2 Cycloastragenol-mediated neuroprotection: from neuroinflammation to cognitive function

Neuroinflammation, as the common pathological basis of various neurological diseases, has always been a hot topic in terms of its regulatory mechanisms and therapeutic strategies. In recent years, CAG has attracted much attention because of its multi-target and multi-pathway anti-inflammatory properties. Its role mechanism in neuroprotection has gradually been revealed, showing a multi-level regulatory network from molecules to systems.

In the pathological process of neurodegenerative diseases, the imbalance between oxidative stress and neurotrophic factor signaling pathways is a key factor leading to neuronal damage. Studies have shown that CAG can effectively reverse the inhibition of the Nrf2/HO-1 pathway induced by Aβ, while activating the signaling axis BDNF/p-TrkB/CREB. This dual effect not only reduces oxidative stress damage and neuroinflammation but also promotes neuronal survival. CAG’s inhibitory effect on p-JNK further blocks the neurotoxicity mediated by the MAPK pathway, which seems to be its main mechanism for inhibiting Aβ-induced MAP kinase activation (Ikram et al., 2021).

Further research has shown that CAG also plays a role in the regulation of microglial polarization. Through its unique bidirectional regulatory mechanism, CAG, on the one hand, inhibits pro-inflammatory polarization of the M1-type mediated by NF-κB and, on the other hand, promotes anti-inflammatory polarization of the M2-type through the Nrf2 pathway. This regulation is reflected not only in the downregulation of proinflammatory factors but also in the upregulation of anti-inflammatory factors and the M2 marker CD206 (Chen et al., 2022). It is particularly noteworthy that CAG’s regulation of neurons and astrocytes is closely related to its antioxidant effects, and the activation of the MAPK/Nrf2/HO-1 pathway may be an important bridge connecting these two major functions (Ying Wu, 2023). In addition, CAG also shows dual functions in the transient focal cerebral ischemia model, with its epigenetic regulatory effects being particularly prominent. By upregulating the expression of SIRT1, CAG achieves dual regulation of p53 and NF-κB: on the one hand, it inhibits neuronal apoptosis through deacetylation of p53, and on the other hand, it reduces neuroinflammation by regulating NF-κB activity. Although the impact of CAG’s on specific acetylation sites of p65 is limited, it significantly reduces the expression of pro-inflammatory factors and overall glial cell activation. In the CAG-treated model, CAG significantly reduces the acetylation of p53 induced by MCAO and reduces the Bax/Bcl-2 ratio in ischemic brain tissue, effectively inhibiting the p53-dependent apoptotic pathway. Although the effect of CAG’s on the acetylation of p65 Lys310 in ischemic brain tissue is not significant, it generally inhibits the activation of NF-κB and reduces the expression of pro-inflammatory cytokines and the activation of glial cells after cerebral ischemia (Li et al., 2020).

Research on the intracerebral hemorrhage model has further expanded our understanding of the neuroprotective mechanisms of CAG’s. CAG can not only improve neurological function in a dose-dependent manner but also works through multiple mechanisms: maintaining the integrity of the blood-brain barrier, reducing brain edema, and regulating the p38-MAPK signaling pathway. It is particularly noteworthy that CAG activation of the Nrf2/HO-1 pathway and inhibition of oxidative stress form a virtuous cycle. This self-reinforcing protective mechanism may be an important reason for its significant therapeutic effects in different models of neurological injury (Ying Wu, 2023).

#### 5.1.3 Cycloastragenol in asthma management: modulation of airway inflammation

The main pathological features of asthma include bronchial hyperresponsiveness (BHR), inflammation of the airways, and excessive mucus secretion (Hammad and Lambrecht, 2021). During the development of asthma, goblet cell hyperplasia and overproduction lead to the formation of airway mucus plugs (Rogers, 2002). CAG can significantly reduce AHR, reduce infiltration of immune cells in the airways, and significantly alleviate the release of pro-inflammatory cytokines, effectively alleviating airway inflammation. Mechanisms include alleviation of AHR through natural killer cell-mediated cytotoxicity (NK), leukocyte transendothelial migration, and regulation of B cell receptor (BCR) and T cell receptor (TCR) signaling pathways (Zhu et al., 2022).

TMT-based quantitative proteomics analysis based on TMT showed that CAG significantly regulates inflammation-related protein networks, especially the integrin αL (ITGAL), spleen tyrosine kinase (Syk), and guanine nucleotide exchange factor Vav1. These proteins play a key role in the activation and migration of T cells, B cells, and NK cells. CAG effectively reduces leukocyte extravasation and adhesion by downregulating ITGAL expression, thereby inhibiting the recruitment of T cells, neutrophils, and NK cells to inflammatory sites. In addition, CAG affects the TCR signaling pathway by inhibiting the expression of Syk and Vav1, thus reducing abnormal leukocyte infiltration during asthma pathogenesis. Syk regulates the recruitment of eosinophils and neutrophils from blood vessels to inflamed tissues, while Vav1 is essential for the development, activation and migration of T cells. CAG also significantly inhibits the ability of inflammatory cells to release pro-inflammatory factors and reduces the production of downstream inflammatory mediators by inhibiting the activation of the p38 MAPK signaling pathway, further alleviating chronic inflammation caused by asthma (Zhu et al., 2022).

In a mouse asthma model, CAG significantly reduced peribronchial inflammatory cell infiltration, with the high-dose treatment group (125 mg/kg) showing the most significant effect. At the same time, CAG significantly reduced the levels of pro-inflammatory cytokines such as IL-5 and IL-13 in bronchoalveolar lavage fluid (BALF), as well as serum immunoglobulin E (IgE) levels, thereby blocking the progression of allergic inflammation and reducing inflammation-induced tissue damage (Zhu et al., 2021).

#### 5.1.4 Cycloastragenol attenuates visceral fibrosis through anti-inflammatory pathways

CAG significantly inhibits visceral fibrosis through various anti-inflammatory mechanisms and demonstrates broad therapeutic potential in studies of cardiac, hepatic, and pulmonary fibrosis.

In cardiac fibrosis, the antifibrotic mechanism of CAG is likely associated with its inhibition of NLRP3 inflammasome activation. Studies have shown that high-dose CAG significantly reduces the expression of Collagen-1 and Collagen-3 mRNA and suppresses increases induced by ISOproterenol (ISO) in cardiac fibrosis (CVF). Although low-dose CAG also inhibits collagen mRNA expression, its inhibitory effect on protein levels is less pronounced than that of high dose CAG, indicating the greater efficacy of high doses in controlling cardiac fibrosis (Wan et al., 2018). CAG also significantly improves liver fibrosis. It effectively alleviates liver fibrosis by regulating the expression of MMPs, reducing pro-fibrotic MMP2 while significantly enhancing the expression of anti-fibrotic MMP8, MMP9 and MMP12. Furthermore, CAG improves IL-6 expression, promoting hepatocyte regeneration after CCl_4_-induced injury, further reducing fibrosis, which is consistent with its key role in liver regeneration (Luangmonkong et al., 2023). In pulmonary fibrosis research, CAG exhibits significant effects by regulating telomerase activity and telomere length. In Tert Het mice treated with CAG, telomerase activity significantly increased, and telomere length moderately extended, accompanied by improvements in lung structure and function, indicating that CAG has potential protective effects against lung tissue aging and fibrosis (Kilic et al., 2024).

#### 5.1.5 Emerging applications of cycloastragenol in other inflammatory conditions

In recent years, oligonucleotides have attracted a great deal of attention as a novel drug carrier due to their unique advantages. Among them, AS1411 has shown great potential as a drug delivery platform due to its excellent targeting ability, high affinity, and low immunogenicity (Hwang et al., 2010; Rosenberg et al., 2013). Against this backdrop, some researchers have successfully conjugated CAG with oligonucleotides to construct a novel ON-CAG conjugate using advanced oligonucleotide synthesis technology. This innovation not only significantly improves the water solubility of CAG but also, to everyone’s delight, shows outstanding renal protective effects in cisplatin induced acute kidney injury (AKI) models. In-depth research has found that the protective mechanism of ON-CAG is closely related to its regulation of oxidative stress and inflammatory responses. In the cisplatin-induced renal injury process, ON-CAG can effectively inhibit ROS overproduction while significantly reducing the expression levels of the renal injury marker KIM-1 and the inflammatory factor IL-18. This dual regulatory effect not only improves the survival rate of renal tubular epithelial cells, but also effectively reduces renal inflammatory damage by downregulating the expression of pro-inflammatory factors such as IL-6 (Tang et al., 2022). These protective effects of ON-CAG are achieved while fully retaining the original biological activity of CAG, which provides important ideas for the development of new renal protective drugs.

More importantly, the regulatory role of CAG is not limited to renal protection. It also shows significant therapeutic potential in cardiovascular diseases. Studies have found that in models of acute myocardial infarction (AMI), CAG exerts cardiac protective effects by regulating the RhoA signaling pathway. As an important GTP-binding protein, overactivation of RhoA can trigger a series of pathological changes: on the one hand, it affects cardiac function by affecting the actin cytoskeleton; on the other hand, it exacerbates inflammatory responses by activating the MAPK family (including p38, MAPK, and ERK1/2). By inhibiting the RhoA/ROCK pathway, CAG not only improves cardiac hypertrophy and remodeling but also reduces inflammatory responses, providing a new target for the treatment of heart failure (Ren et al., 2020).

**FIGURE 4:**
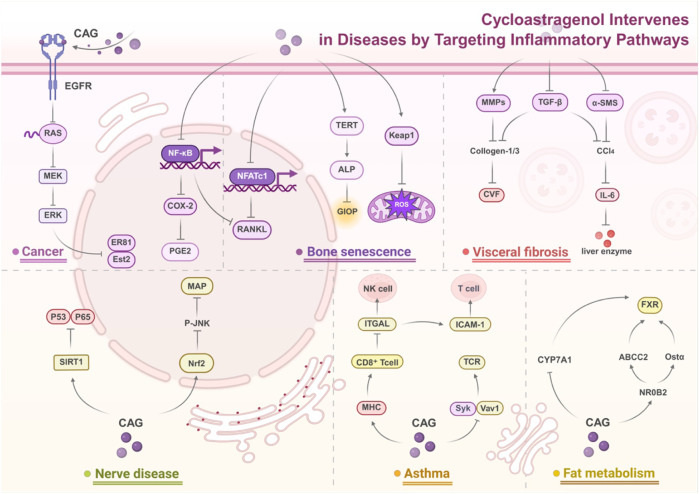
Cycloastragenol Intervenes In diseases by targeting inflammatory pathways.

In summary, existing studies suggest that CAG regulates systemic cellular inflammatory responses through multiple pathways, encompassing a wide range of applications from renal protection to the treatment of cardiovascular disease. These findings not only deepen the understanding of CAG’s anti-inflammatory mechanisms but also provide critical theoretical support for its clinical application in various inflammation related diseases. Despite the promising therapeutic effects of CAG in various inflammation-related diseases, several scientific and technical challenges need to be addressed to translate its potential into clinical applications. The safety and efficacy of CAG in human disease treatment have not yet been fully validated. The potential side effects and long-term results associated with prolonged use of CAG remain unclear, individual responses to CAG may differ. Future research should consider these individual differences to achieve precision medicine. More detailed studies are needed on the design of clinical trials, drug development, and personalized treatment approaches (Figure 4).

### 5.2 Cycloastragenol: research progress and translation in multifield clinical applications

#### 5.2.1 Cycloastragenol-mediated regulation of metabolism *via* FXR signaling

Research on the mechanism of action of CAG in the treatment of metabolic diseases has revealed its multiple regulatory effects through the farnesoid X receptor (FXR) signaling pathway. As an important member of the nuclear receptor family, the activation of FXR constitutes the core mechanism of CAG in improving metabolic disorders. Studies have shown that CAG can directly bind to and activate FXR in a dose-dependent manner, thereby regulating the expression of a series of downstream target genes. This indicates that CAG may act as a potential FXR agonist by directly binding to FXR and enhancing its transcriptional activity (Gu et al., 2017). FXR agonists have been shown to have significant therapeutic effects on non-alcoholic fatty liver disease (NAFLD), and CAG can effectively activate FXR, reduce liver fibrosis, and decrease hepatocyte apoptosis. This effect has shown significant therapeutic effects in multiple metabolic-related disease models (Wang et al., 2024c).

In terms of regulation of bile acid metabolism, CAG establishes a complete negative feedback regulation loop of bile acids by upregulating the expression of FXR target genes such as NR0B2 and FGF19, while inhibiting the activity of the rate-limiting enzyme for the synthesis of bile acids, CYP7A1. This regulation not only improves the metabolic balance of bile acids, but also significantly alleviates hepatic lipid accumulation by activating the FXR-PPARα signaling axis. In diet-induced obesity models, this dual regulatory effect of CAG is particularly prominent: it reduces bile acid synthesis by decreasing the expression of hepatic Cyp7A1 and Cyp8B1 and improves glucose metabolic disorders by inhibiting the expression of key gluconeogenic enzymes such as Pck1 and G6pc. CAG-activated FXR activation shows tissue-specific characteristics. In intestinal tissue, by upregulating the expression of target genes such as Nr0b2 and Ostα, CAG may be involved in the regulation of enterohepatic circulation, providing a new perspective to explain its role in improving metabolic syndrome. In models of non-alcoholic steatohepatitis, CAG not only reduces hepatic steatosis but also inhibits the progression of liver fibrosis by decreasing the expression of CK19 and α-SMA, which are likely achieved through FXR-mediated anti-inflammatory and anti-fibrotic effects (Gu et al., 2017).

In addition to the FXR pathway, CAG also participates in the liver regeneration process by regulating the expression of AQP9. As a key protein for maintaining liver metabolic homeostasis, the activation of AQP9 promotes the uptake of glycerol and gluconeogenesis in the liver, while reducing oxidative stress damage by enhancing the efflux of H_2_O_2_. This dual metabolic-regenerative regulatory mechanism provides important clues for understanding the role of CAG in promoting liver repair and suggests that there may be an as-yet-unelucidated interaction between FXR signaling and AQP9 function (Li et al., 2024).

#### 5.2.2 Cycloastragenol in bone homeostasis: dual modulation of osteoclastogenesis and osteoblast differentiation

Research on the mechanism of action of CAG in the treatment of osteoporosis has revealed its multi-level regulatory network for bone metabolic balance. The core pathology of osteoporosis lies in the imbalance between excessive osteoclast activation and insufficient osteoblast function, and CAG shows unique therapeutic advantages by targeting these two key aspects.

In terms of osteoclast regulation, CAG exhibits multiple mechanisms to inhibit differentiation from the source. Osteoclast precursors originate from the mononuclear/macrophage system, and their differentiation and maturation depend on the sequential activation of two key signaling pathways: M-CSF/c-FMS and RANK/RARK. Studies have found that CAG can dose-dependently block the differentiation of bone marrow macrophages into osteoclasts induced by RANKL. This effect is achieved by simultaneously inhibiting NF-κB signaling, calcium signaling pathways, and NFATc1 activation. CAG also alleviates oxidative damage in bone tissue by activating the Nrf2/Keap1/ARE antioxidant pathway. This dual protection mechanism significantly mitigates postmenopausal bone loss in the ovariectomized model (Buder-Hoffmann et al., 2009; Wang G. et al., 2024).

For glucocorticoid-induced osteoporosis (GIOP), CAG exerts its therapeutic effect by promoting osteoblast differentiation. As a telomerase activator, CAG significantly enhances alkaline phosphatase (ALP) activity and mineralization capacity by upregulating TERT expression, while also promoting the expression of various osteogenic-related factors, including Runx2. This mechanism has been verified in both cell experiments and in zebrafish models. In particular, when the telomerase inhibitor TMPyP4 is used to block TERT, the protective effect of CAG disappears, fully demonstrating the central role of telomerase activation in its anti-GIOP effect (Wu et al., 2020).

In age-related osteoporosis, the therapeutic mechanism of CAG is more comprehensive. By upregulating osteoactivin (OA) expression, CAG not only promotes osteogenic differentiation of mesenchymal stem cells (MSCs), but also directly inhibits osteoclast activity. This dual regulatory effect significantly improves bone microstructure and biomechanical properties. Proteomics analysis further reveals that OA, as a type I transmembrane glycoprotein, can significantly enhance the osteogenic differentiation potential of MSCs and maintain bone metabolic balance *in vivo* by promoting bone formation and inhibiting bone resorption. By inducing OA expression, CAG can slow down age-related bone loss and significantly improve bone microstructure and biomechanical properties (Yu et al., 2020).

**FIGURE 5:**
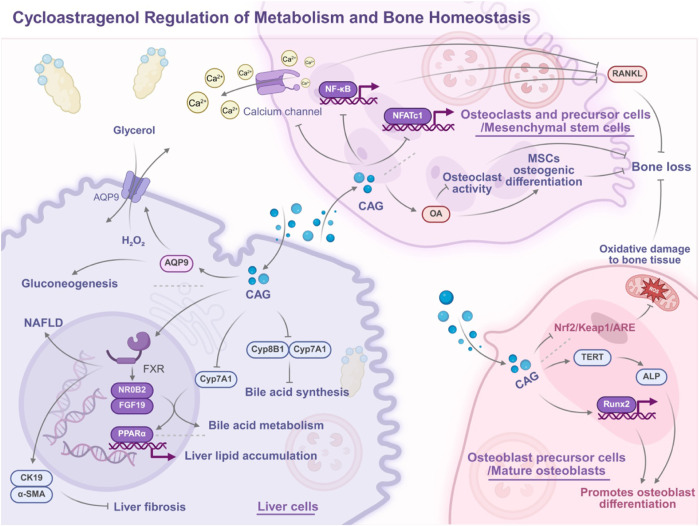
Mechanisms of cycloastragenol regulation of metabolism and bone homeostasis.

CAG holds potential in preventing and treating metabolism disorders and osteoporosis. While preliminary studies suggest that its mechanisms of action are related to the signaling pathways of FXR and Nrf2/Keap1/ARE, specific mechanisms and its interactions with other molecules require further investigation. The synergistic effects of CAG with other drugs or therapies need to be further examined to improve efficacy and reduce side effects. In addition, as a natural product, CAG has high extraction and purification costs, which limits its clinical application. Therefore, reducing production costs is crucial (Figure 5).

## 6 Toxicological profile of cycloastragenol

The toxicological profile of CAG has been thoroughly evaluated in both clinical and preclinical settings, revealing an overall favorable safety margin under diverse experimental conditions. However, the dose-dependent toxicities observed *in vitro* underscore the need for more precise assessments to establish its therapeutic window. This section integrates findings from clinical trials, animal studies, and *in vitro* experiments to provide a cohesive perspective on the toxicological properties of CAG, highlighting both its strengths and areas requiring further investigation.

### 6.1 Cycloastragenol clinical toxicological evaluation

Initially, the safety of CAG has been sufficiently validated in numerous preclinical animal studies, with its safety profile showing dose dependence and universality of the model. In terms of basic toxicity assessment, a 33-week oral administration study in rats conducted by Yu et al. demonstrated that no adverse events, behavioral abnormalities, or pathological changes were observed even at high exposure levels (Yu et al., 2020). In particular, CAG not only possesses intrinsic safety, but also exhibits organ-protective properties: Luangmonkong et al. found that a dose of 200 mg/kg of CAG was non-toxic to the liver and significantly alleviated CCl4-induced liver injury in mice (Luangmonkong et al., 2023), while Chen et al. confirmed that 62.5 mg/kg of CAG effectively increased the survival rate of septic mice (Chen et al., 2025a). Additionally, studies targeting specific age groups have shown that a daily dose of 150 mg/kg of TA65 (a CAG-related substance) was well-tolerated in adult and aged female mice over a 4-month observation period, as reported by the Salvador team (Salvador et al., 2016). Collectively, these studies have constructed a safety evidence system for CAG, with data covering a broad dose range of 25–200 mg/kg and varying experimental durations of 4–33 weeks. The good safety margin demonstrated across multiple animal models and dosing conditions provides a solid experimental basis for subsequent clinical translation.

In terms of genotoxicity, standard assays have shown that CAG is non-mutagenic. The bacterial reverse mutation assay revealed that CAG did not have mutagenic activity against *Salmonella typhimurium* and *Escherichia coli* strains at doses up to 5,000 μg per plate. Similarly, the *in vivo* micronucleus test in mice administered 2,000 mg·kg^−1^ of CAG intraperitoneally showed no pathogenic effects on red blood cells, further confirming its genomic safety (Szabo, 2014). Although these results confirm that CAG does not pose mutagenic or teratogenic risks under experimental conditions, the lack of long-term studies of carcinogenicity is a key area for future research. These findings highlight the importance of dose-dependent assessment, especially in terms of cell-specific vulnerabilities and their relevance to high-dose clinical scenarios.

Furthermore, the results of the clinical study are consistent with animal studies, showing a low toxicity of CAG under the recommended dosing regimen. A 1-year clinical trial (daily intake of 32 mg of TA-65) reported no drug-related adverse events, indicating excellent tolerability of CAG with long-term use, which is consistent with its widespread use as a health supplement in the general population (Salvador et al., 2016). The Paton-1 protocol, a business program aimed at improving health, which involved approximately 7,000 participants receiving a supplement package containing CAG for 5 years, has been shown to indicate no discomfort or adverse effects from taking this supplement package (Harley et al., 2013). *In vitro* studies have shown that CAG has dose-dependent cytotoxicity. Many cell lines (such as osteoblast precursors) exhibited good tolerance to CAG concentrations up to 3 μM within 72 h (Yu et al., 2020), but certain cell types showed significant cytotoxic effects at higher concentrations. For instance, it exerts significant cytotoxic effects on primary cardiac fibroblasts when the concentration of cycloastragenol exceeds 100 mg/L, whereas no cytotoxicity is observed at concentrations below 62.5 mg/L (Wan et al., 2018).

Regarding the initial toxicity threshold, this finding is from a 91-day subchronic toxicity study by Szabo, in which a daily dose of 150 mg/kg of CAG did not induce treatment-related mortality or adverse reactions in either male or female rats (NOAEL was 150 mg/kg/day, suggesting a tolerable daily intake for humans of ≤105 mg) (Szabo, 2014). The *in vitro* inhibition IC_50_ of UGT in humans is as low as 0.84 µM. If a single oral dose in humans is too high or taken too long, relevant indicators must be monitored (Ran et al., 2016). In summary, CAG is safe for oral doses ≤105 mg/day, with a tentative therapeutic window of maximum blood concentration of 0.05–1 μM, daily dose ≤100 mg. Exceeding the upper limit requires evaluation of drug interactions and liver function. However, the lack of longer-term and higher-dose human safety data remains a key gap, especially for therapeutic applications beyond dietary supplements. Addressing this limitation is crucial to ensure its safe use in pharmacological doses.

### 6.2 Future directions for safety assessment

**FIGURE 6:**
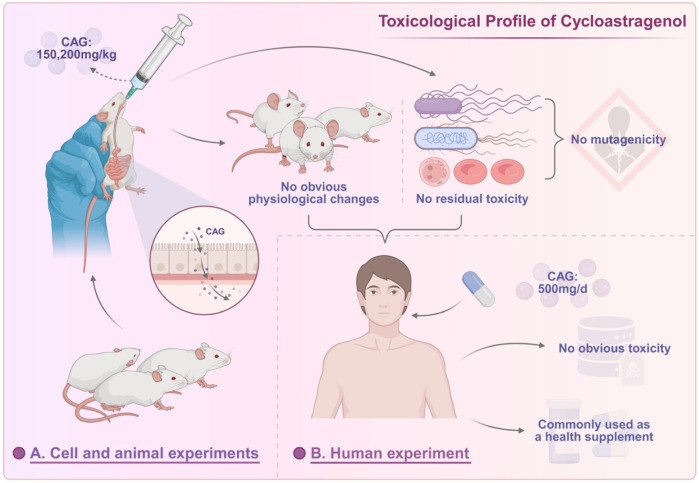
Toxicological profile of cycloastragenol.

Based on the aforementioned clinical data, although the toxicological profile of CAG is generally favorable (Figure 6), there are still some challenges to ensure the safe and effective use of CAG in clinical practice. To begin with, the cytotoxic effects observed at high concentrations warrant a detailed investigation to delineate their precise therapeutic index. Mechanistic studies exploring the roles of oxidative stress and mitochondrial dysfunction in its dose-dependent toxicity are particularly critical. Second, the absence of data on long-term carcinogenicity and reproductive toxicity underscores the need for extended studies to evaluate its safety in chronic use. Lastly, inter-individual variability in metabolism, driven by genetic polymorphisms or gut microbiota composition, may influence toxicity risks. Addressing these gaps through advanced toxicological modeling and bioanalytical tools will be essential to optimize their clinical application. Collectively, CAG exhibits a low toxicity profile in a variety of experimental conditions, supporting its potential as both a therapeutic agent and a health supplement. However, the dose-dependent toxicities observed *in vitro* highlight the need to refine its therapeutic index and perform long-term safety studies. Future research should focus on elucidating its molecular mechanisms of toxicity, investigating its long-term safety profile, and addressing inter-individual differences to facilitate its broader adoption in clinical settings.

## 7 Conclusions and perspectives

### 7.1 Summary of pharmacological effects and therapeutic potential of cycloastragenol

CAG is a bioactive triterpenoid compound derived from *Astragalus membranaceus*. Its unique dual capability allows it to simultaneously regulate oxidative stress and inflammation *via* the Nrf2/HO-1 and NLRP3 inflammasome pathways, thereby distinguishing it from traditional anti-inflammatory agents (Chen et al., 2022; Wan et al., 2018). Unlike synthetic drugs that typically target a single pathway (e.g., COX-2 inhibitors or glucocorticoids), CAG orchestrates a self-reinforcing cellular protection network. It exerts direct anti-inflammatory effects by inhibiting NF-κB and MAPK signaling (Chen et al., 2025a; Xiao et al., 2025), performs ROS scavenging activity through Nrf2-mediated induction of antioxidant genes (Wang et al., 2022b; Ying Wu 2023), and addresses chronic inflammation by AMPK and SIRT1 activation (Li et al., 2020; Lin et al., 2022; Zhu et al., 2024). This triple mechanism is the basis for its broad therapeutic efficacy in diseases in which inflammation and oxidative pathology are intertwined. For example, in neurodegeneration, it crosses the BBB to inhibit microglial pyroptosis (Wu et al., 2023), regulates TGF-β/pSmad3 and MMP in anti-fibrosis (Luangmonkong et al., 2023), and modulates metabolic disorders through FXR-dependent regulation of bile acids (Wang et al., 2024c). CAG’s telomerase-activating property, i.e., upregulation of TERT, expands its therapeutic scope from symptom relief to disease modification, delaying cellular senescence in osteoporosis and cardiovascular aging (Kilic et al., 2024; Wu et al., 2020). Its low toxicity and gut microbiota-dependent bioavailability further position it as a prototype for the next-generation of nutraceutical-drug hybrids (Hu et al., 2023; Jin et al., 2015; Yu et al., 2018). However, CAG’s “natural drug advantage,” multi-pharmacology with minimal off-target effects, also presents translational challenges, requiring precise delivery systems (such as oligonucleotide conjugates) to fully exploit its potential (Tang et al., 2022).

### 7.2 Challenges in clinical application of cycloastragenol

Despite its promising pharmacological activities, the clinical translation of CAG faces several challenges. Primarily, its precise mechanisms of action remain incompletely elucidated. Although evidence suggests CAG modulates multiple signaling pathways and cytokines, the interplay between these mechanisms and their disease-specific roles requires deeper investigation. In addition, the selective modulation of inflammatory pathways in different cell types and tissues remains poorly understood. The research findings indicate that CAG deactivates inflammatory and apoptotic pathways by blocking the upregulation of SphK, MIP-1α, and NF-κB expression, as well as the downregulation of miR-143 expression induced by ulcerative colitis (Bagalagel et al., 2022). Moreover, qRT-PCR and Western blot analyses reveal that CAG can regulate the proliferation and autophagy of keratinocytes stimulated by IL-22 (Xia et al., 2024). However, the interplay among these distinct mechanisms still needs to be explored in more detail. Future studies could combine cellular and animal models, such as using LPS induced BV2 cells to construct an *in vitro* neuroinflammatory model (Xiao et al., 2025), Hb-induced subarachnoid hemorrhage models (Lin et al., 2022), and OVA-induced asthma mouse models (Zhu et al., 2021), to further elucidate the mechanisms of action of CAG. Integrating multidisciplinary research methods from chemistry, biology, and pharmacology will help to comprehensively reveal the molecular mechanisms of CAG in various diseases and provide a solid theoretical basis for its clinical application.

In addition, the bioavailability of CAG is notably low, constrained by poor solubility and extensive first-pass metabolism, which limits its effectiveness *in vivo* concentrations. For instance, studies in rats reveal an oral bioavailability of only 25.70% (Ma et al., 2016), necessitating high doses to achieve therapeutic effects. Addressing this limitation is critical, with research prioritizing advanced drug delivery systems such as nanoparticles, liposomes, or targeted formulations to enhance bioavailability and tissue-specific targeting. Studies have also shown that coupling CAG with oligonucleotides can significantly enhance its water solubility and renal protective effects (Tang et al., 2022). Additionally, the scarcity of clinical studies represents a significant bottleneck. Large-scale, multicenter clinical trials are essential to validate its dose-dependent efficacy, safety, and therapeutic potential. Notably, high concentrations of CAG have exhibited cytotoxicity in certain cell types, raising concerns about potential adverse effects in clinical applications (Wan et al., 2018). Thus, rigorous preclinical and clinical evaluations are imperative to optimize its pharmacological profile and establish rational treatment protocols for indications such as neurodegenerative diseases and diabetic complications.

### 7.3 Emerging research Horizons of cycloastragenol

Future research should prioritize elucidating the molecular mechanisms of CAG, optimizing drug formulations, and advancing clinical applications. Integrating gene editing technologies, omics-based analyzes, and pharmacological experiments could uncover key molecular targets within inflammatory signaling pathways and their interactions with intracellular networks. For instance, CAG has been shown to promote cardiomyocyte autophagy *via* the AKT1-RPS6KB1 pathway, inhibit NF-κB activation to reduce pro-inflammatory cytokine production, and enhance HO-1 activity to suppress inflammatory responses (Wang et al., 2018a). Currently, the development of innovative drug delivery systems, such as nanoparticle-based formulations or oligonucleotide conjugates, could significantly improve bioavailability and therapeutic targeting. On the clinical front, designing large-scale multicenter trials is critical to establish its safety, efficacy, and optimal dosing regimens in various diseases. Additionally, exploring synergistic combination therapies, such as pairing CAG with chemotherapeutic agents such as 5-FU or paclitaxel, holds promise to improve anticancer efficacy while minimizing adverse effects associated with monotherapy (Park et al., 2022). The establishment of stringent quality control and standardization protocols is another crucial step toward clinical translation. Given the natural origin of CAG, its pharmacological activity may vary according to cultivation conditions and extraction processes. Employing advanced quality monitoring technologies can ensure consistent, safe, and stable formulations. For example, employing online near-infrared spectroscopy probes to monitor the concentration changes of active ingredients in the extract in real-time ensures the efficiency and stability of the extraction process. Meanwhile, the use of multivariate statistical process control (MSPC) technology strictly guaranties the consistency of the samples. In addition, ultrasonic extraction and microwave extraction technologies, known for their high efficiency, environmental friendliness, and selective extraction capabilities, have garnered significant attention. The application of these advanced technologies will greatly facilitate the establishment of a quality control and standardization system for CAG, thereby ensuring the stability and safety of product quality.

**FIGURE 7:**
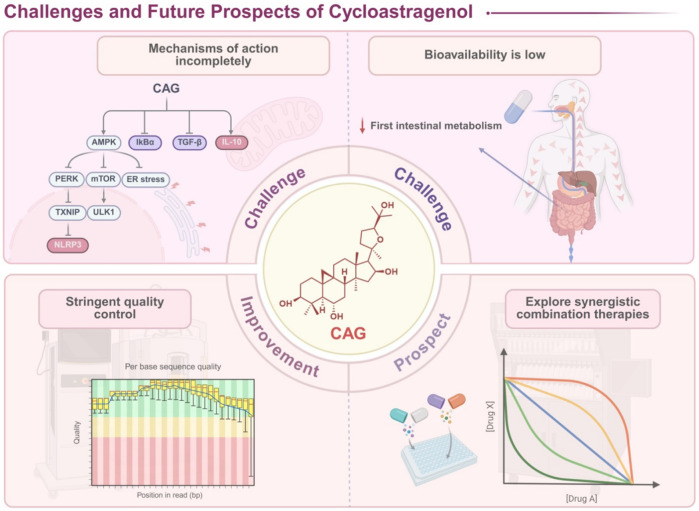
Conclusions and perspectives of cycloastragenol.

In conclusion, CAG, a natural compound with various pharmacological properties, has demonstrated significant therapeutic potential in preclinical studies of inflammation-related diseases. However, translating this potential into effective clinical applications requires addressing key challenges, including elucidating its mechanisms of action, improving bioavailability, and validating clinical efficacy. Through multidisciplinary collaboration and technological innovation, these obstacles can be overcome, paving the way for CAG to become a safe, low-toxicity, and highly effective therapeutic agent (Figure 7).

## Glossary

CAG: cyclastragenol
NSAIDs: non-steroidal anti-inflammatory drugs
MVA: mevalonate
MEP: methylerythritol phosphate
IPP: isopentenyl diphosphate
DMAPP: dimethylallyl diphosphate
FPS: farnesyl pyrophosphate synthase
FPP: farnesyl pyrophosphate
SS: squalene synthase
SE: squalene epoxidase
OSC: oxidosqualene cyclase
CYP450s: cytochrome P450 enzymes
CA-2H: dehydroCAG
iso-CA: isoCAG
CAS: cycloartenol synthase
Cmax: peak plasma concentration
T1/2: halflife
AUC: area under the curve
UGT: UDP-glucuronosyltransferase
AST: astragaloside IV
ISO: isoprenaline
NF-κB: factor κb
TGF-β: factor β
EMT: epithelial-mesenchymal transition
Nrf2: nuclear factor erythroid 2-related factor 2
ROS: oxygen species
Th: T helper
PAMPs: pathogen-associated molecular patterns
DAMPs: damage-associated molecular patterns
LPS: lipopolysaccharide
IMQ: imiquimod
AMPK: AMP-activated protein kinase
p38-MAPK: p38 mitogen-activated protein kinase
NSCLC: non-small cell lung cancer
TXNIP: thioredoxin-interacting protein
TNF-α: tumor necrosis factor-α
IL-1β: interleukin-1β
VSMCs: vascular smooth muscle cells
MMPs: matrix metalloproteinases
ECM: extracellular matrix
CQ: Chloroquine
MHC-I: major histocompatibility complex class I
Con-A: concanavalin A
IL-17A: Th17-type cytokines
GZMB: granzyme B
AAA: abdominal aortic aneurysm
PGE2: prostaglandin E2
TAM: tumor-associated macrophage
TERT: telomerase reverse transcriptase
BDNF: brain-derived neurotrophic factor
MCAO: middle cerebral artery occlusion
BBB: blood-brain barrier
BWC: brain water content
NK: natural killer
BCR: B cell receptor
TCR: T cell receptor
ITGAL: integrin αl
Syk: spleen tyrosine kinase
TRACP: tartrate-resistant acid phosphatase
BALF: bronchoalveolar lavage fluid
IgE: immunoglobulin E
ISO: isoproterenol
CVF: cardiac fibrosis
AKI: acute kidney injury
AMI: acute myocardial infarction
FXR: farnesoid X receptor
NAFLD: nonalcoholic fatty liver disease
NASH: nonalcoholic steatohepatitis
HF: high-fat
NFATc1: nuclear factor of activated T cells 1
OVF: ovariectomized
GIOP: Glucocorticoid-induced osteoporosis
ALP: alkaline phosphatase
DEX: dexamethasone
OA: osteoactivin
MSCs: mesenchymal stem cells
MSPC: multivariate statistical process control
